# Indian Antimicrobial Prescription Guidelines in Critically Ill Immunocompromised Patients

**DOI:** 10.5005/jp-journals-10071-23102

**Published:** 2019-01

**Authors:** Atul P Kulkarni,, Manju Sengar,, Girish Chinnaswamy,, Ashit Hegde,, Camilla Rodrigues,, Rajeev Soman,, Gopi C Khilnani,, Suresh Ramasubban,, Mukesh Desai,, Rahul Pandit,, Ruchira Khasne,, Anjali Shetty,, Trupti Gilada,, Shilpushp Bhosale,, Amol Kothekar,, Subhal Dixit,, Kapil Zirpe,, Yatin Mehta,, Jacob George Pulinilkunnathil,, Vikas Bhagat,, Mohammad Saif Khan,, Amit M Narkhede,, Nishanth Baliga,, Srilekha Ammapalli,, Shrirang Bamne,, Siddharth Turkar,, Vasudeva Bhat K,, Jitendra Choudhary,, Rishi Kumar,, Jigeeshu V Divatia

**Affiliations:** 1 Division of Critical Care Medicine, Department of Anaesthesiology, Critical Care and Pain, Tata Memorial Hospital, Homi Bhabha National Institute, Dr Ernest Borges Road, Parel, Mumbai, Maharashtra, India; 2 Department of Medical Oncology, Tata Memorial Centre, Homi Bhabha National Institute, Dr Ernest Borges Road, Parel, Mumbai, Maharashtra, India; 3 Department of Paediatric Oncology, Tata Memorial Centre, Homi Bhabha National Institute, Dr Ernest Borges Road, Parel, Mumbai, Maharashtra, India; 4 Consultant in Medicine and Critical Care, PD Hinduja National Hospital, Mahim, Mumbai, Maharashtra, India; 5 Consultant Microbiologist and Chair Infection Control, Hinduja Hospital, Mahim, Mumbai, Maharashtra, India; 6 Consultant ID Physician, Jupiter Hospital, Pune, DeenanathMangeshkar Hospital, Pune, BharatiVidyapeeth, Deemed University Hospital, Pune, Courtsey Visiting Consultant, Hinduja Hospital Mumbai, Maharashtra, India; 7 Department of Pulmonary Medicine and Sleep Disorders, All India Institute of Medical Sciences, New Delhi, India; 8 Pulmomary and Critical Care Medicine, Apollo Gleneagles Hospital, 58, Canal Circular Road, Kolkata, West Bengal, India; 9 Department of Immunology, Prof of Pediatric Hematology and Oncology, Bai Jerbaiwadia Hospital for Children, Consultant, Hematologist, Nanavati Superspeciality Hospital, Director of Pediatric Hematology, Surya Hospitals, Mumbai, Maharashtra, India; 10 Intensive Care Unit, Fortis Hospital, Mulund Goregaon Link Road, Mulund (W), Mumbai, Maharashtra, India; 11 Critical Care Medicine, Ashoka - Medicover Hospital, Indira Nagar, Wadala Nashik, Maharashtra, India; 12 Microbiology Section, 5th Floor, S1 Building, PD Hinduja Hospital, Veer Savarkar Marg, Mahim, Mumbai, Maharashtra, India; 13 Consultant Physician in Infectious Disease, Unison Medicare and Research Centre and Prince Aly Khan Hospital, Maharukh Mansion, Alibhai Premji Marg, Grant Road, Mumbai, Maharashtra, India; 14 Intensive Care Medicine, Department of Anaesthesia, Critical Care and Pain, Tata Memorial Center, Homi Bhabha National Institute, Dr. E. Borges Road, Parel, Mumbai, Maharashtra, India; 15 Division of Critical Care Medicine, Departemnt of Anaesthesia, Critical Care and Pain, Tata Memorial Center, Homi Bhabha National Institute, Dr. E. Borges Road, Parel, Mumbai, Maharashtra, India; 16 Consultant in Critical Care, Director, ICU Sanjeevan and MJM Hospital, Pune, Maharashtra, India; 17 Neuro-Trauma Unit, Grant Medical Foundation, Ruby Hall Clinic, Pune, Maharashtra, India; 18 Institute of Critical Care and Anesthesiology, Medanta The Medicity, Gurgaon, Haryana, India; 19 Division of Critical Care Medicine, Department of Anesthesia, Critical Care and Pain, Tata Memorial Hospital, Homi Bhabha National Institute, Dr E Borges Road, Mumbai, Maharashtra, India; 20 Department of Anaesthesia, Critical Care and Pain, Tata Memorial Center, HomiBhabha National Institute, Dr. E. Borges Road, Parel, Mumbai, Maharashtra, India; 21 Department of Anaesthesia, Critical Care and Pain, Tata Memorial Center, Homi Bhabha National Institute, Dr. E. Borges Road, Parel, Mumbai, Maharashtra, India; 22 Division of Critical Care Medicine, Department of Anaesthesia, Critical Care and Pain, Tata Memorial Center, Homi Bhabha National Institute, Dr. E. Borges Road, Parel, Mumbai, Maharashtra, India; 23 Division of Critical Care Medicine, Department of Anaesthesia, Critical Care and Pain, Tata Memorial Center, Homi Bhabha National Institute, Dr. E. Borges Road, Parel, Mumbai, Maharashtra, India; 24 Division of Critical Care Medicine, Department of Anaesthesia, Critical Care and Pain, Tata Memorial Center, Homi Bhabha National Institute, Dr. E. Borges Road, Parel, Mumbai, Maharashtra, India; 25 Division of Critical Care Medicine, Department of Anaesthesia, Critical Care and Pain, Tata Memorial Center, Homi Bhabha National Institute, Dr. E. Borges Road, Parel, Mumbai, Maharashtra, India; 26 Department of Medical Oncology, Tata Memorial Hospital, HomiBhabha National Institute, Mumbai, Maharashtra, India; 27 Department of Pediatric Oncology, Tata Memorial Hospital, HomiBhabha National Institute, Dr E. Borges Marg, Parel, Mumbai, Maharashtra, India; 28 Critical Care, Fortis Hospital, 102, Nav Sai Shakti CHS, Near Bhoir Gymkhana, M Phule Road, Dombivali West Mumbai, Maharashtra, India; 29 Critical Care Medicine, PD Hinduja National Hospital and MRC, Mumbai, Maharashtra, India; 30 Department of Anaesthesia, Critical Care and Pain, Tata Memorial Hospital, Homi Bhabha National Institute, Dr. E. Borges Road, Parel, Mumbai, Maharashtra, India

**Keywords:** Asplenia, Febrile neutropenia, Human immunodeficiency virus, Immunocompromised patients, Primary immunodeficiency disorders, Solid organ transplant

## Abstract

**How to cite this article:** Kulkarni AP, Sengar M, Chinnaswamy G, Hegde A, Rodrigues C, Soman R, Khilnani GC, Ramasubban S, Desai M, Pandit R, Khasne R, Shetty A, Gilada T, Bhosale S, Kothekar A, Dixit S, Zirpe K, Mehta Y, Pulinilkunnathil JG, Bhagat V, Khan MS, Narkhede AM, Baliga N, Ammapalli S, Bamne S, Turkar S, Bhat KV, Choudhary J, Kumar R, Divatia JV. Indian Journal of Critical Care Medicine 2019;23(Suppl 1): S64-S96.

## INTRODUCTION

The number of admissions of immunocompromised patients in the Indian intensive care units (ICUs) is growing. This is because of availability of better treatments for acquired immunodeficiency states, increasing incidence and detection of cancer, with more aggressive therapies aimed at cancer cure, and increased expectations of better ICU outcomes in the cancer patient.^1,2^

The indications of immunosuppression have expanded, contributing to an increased number of immunocompromised patients. There is a sharp rise in the number of solid organ transplants being performed in India contributing to the increased number of patients with immunosuppression getting admitted to the ICU in immediate postoperative and subsequent follow-up period. In the pediatric population, we can recognize the genetic predisposition of patients to congenital immunodeficiency states. All these patients have greater susceptibility to new infections or reactivation of latent infections.^3^ Therefore, it is the need of the hour to develop Indian guidelines for prescribing antimicrobial therapy in this population presenting to the ICU.

We feel that the outcomes are likely to be better if these immunocompromised patients with infectious diseases requiring ICU admission are preferably treated at a tertiary care center where all diagnostic facilities along with specialists in microbiology, immunology, infectious disease are likely to be available. We present guidelines in five separate sections: febrile neutropenic patients, patients who have undergone a solid organ transplant, patients with human immunodeficiency virus infections presenting to ICU, congenital asplenia or hyposplenia, and those with congenital primary immunodeficiency syndromes.

These guidelines were developed starting with a collection of Indian and other data which was communicated to members by electronic communication. A meeting of all committee members was held where the data was presented in the form of questions pertaining to the evidence, available evidence from Indian ICUs, and evidence statements and recommendations. A draft of each section was prepared and circulated and finally presented in another committee meeting. The final draft of guidelines was prepared and again communicated to members. The GRADE system was used for the quality of evidence and recommendations (Table 1).

**Table 1:** Quality of evidence and recommendations

*Quality of Evidence*	*Level of evidence*
Evidence from ≥ 1 good quality and well conducted randomized control trial(s) or meta-analysis of RCT's.	1
Evidence from at least 1 RCT of moderate quality, or well-designed clinical trial without randomization; or from cohort or case-controlled studies.	2
Evidence from descriptive studies, or reports of expert committees, or opinion of respected authorities based on clinical experience.	3
Not backed by sufficient evidence; however, a consensus reached by the working group, based on clinical experience and expertise.	Useful Practice Points (UPP)

**Table:** 

*Strength of Recommendation*	*Grade*
Strong recommendation to do (or not to do) where the benefits clearly outweigh the risk (or vice versa) for most, if not all patients.	A
Weak recommendation, where benefits and risk are more closely balanced or are more uncertain.	B

## PART 1. PATIENT WITH FEBRILE NEUTROPENIA IN THE INTENSIVE CARE UNIT

Febrile neutropenia (FN) is defined as an oral temperature of >38.3° C or two consecutive readings of > 38.0° C for 2 hours and an absolute neutrophil count (ANC) of < 0.5 × 10^9^/L or expected to fall below 0.5 × 10^9^/L.^4^

These guidelines are applicable to a critically ill febrile neutropenic patient presenting to the ICU, with any of the following clinical or laboratory parameters of organ failure but not limited to:

- Hypotension
- Tachypnoea requiring oxygen therapy more than 4 liters/minute to maintain saturation > 90%
- Altered mental status (without focal neurological deficit)
- Oliguria or rising creatinine

### Empiric Antibiotic Therapy in Critically Ill Febrile Neutropenic Patients with Suspected Bloodstream Infection

### Evidence Statements

- In a critically ill febrile neutropenic patient presenting to the ICU with organ failure, empiric antibiotic therapy should be initiated or escalated to a broad spectrum carbapenem like imipenem or meropenem (UPP, A).
- Empiric combination of Imipenem or Meropenem and Colistin/Polymyxin B may be considered in the following patients having a high risk of infection with resistant gram-negative organisms (UPP, A):
  - Critically ill patients with underlying acute Leukaemia presenting to the ICU.
  - Patients of Leukaemia/Lymphoma on β-lactam/β-lactamase inhibitor (BL/BLI) ± aminoglycosides, shifted to ICU from the ward.
  - Previous exposure to broad-spectrum antibiotics like carbapenem or BL/BLI combination in last 1 month.
  - Hypotensive patients requiring vasopressor infusions (refractory septic shock).
  - Patient shifted to the ICU on carbapenem therapy.
- We strongly caution against the use of an empiric combination of Imipenem or Meropenem and Colistin/Polymyxin B or Colistin/Polymyxin B alone in patients who are not a high risk of infection with carbapenem-resistant gram-negative organisms as defined above (UPP, A).
- We recommend against empirical use of Doripenem and Ertapenem (III, A)
- Vancomycin/Teicoplanin should be added as empiric therapy in a critically ill febrile neutropenic patient with
  - Suspected Indwelling vascular catheter infection
  - Skin and soft-tissue infection
  - Previous Colonization/infection with methicillin-resistant Staphylococcus aureus (MRSA)
  - Blood Culture: Gram-positive cocci awaiting identification
  - Severe mucositis (III, A)
  - Hemodynamic instability (hypotension) in patients admitted from home/OPD (UPP, A)
- We caution against empirical use of Vancomycin in another group of patients including patients on broad-spectrum antibiotics admitted to the ward and presenting to ICU with hemodynamic instability unless there is a high incidence of MRSA in the institute (UPP, A)

### Evidence Summary

#### Common isolates in a blood culture in febrile neutropenic patients

In India, in febrile neutropenic patients, gram-negative bacteremia is much more common than gram-positive bacteremia (Table 2); in contrast to the western data, where gram-positive isolates are more common.^5,6^ The spectrum of bacterial isolates from a number of studies in India suggest *Enterobacteriaceae* (*E. coli* and *Klebsiella* species) and *Pseudomonas aeruginosa* be the most common among gram-negative organisms. Among gram-positive isolates, *Staphylococcus aureus* and Coagulase-negative *staphylococcus* (CoNS) are most common isolates.

There are scarce data regarding the choice of empirical antibiotic regimens in critically ill febrile neutropenic presenting to the Indian ICUs. Most of the studies have a heterogeneous patient population–leukemia, lymphoma, solid, etc. Choice of antibiotics depends on the most likely causative microorganism, local antimicrobial sensitivity patterns, mechanism of action of antimicrobials, and site of infection, patient's predisposing condition and treating physician's judgment. Recent data show an increased prevalence of Multidrug-resistant (MDR) organisms. Several studies in India have shown that the majority of gram-negative bacteria isolated on initial blood cultures from patients were resistant to the non-carbapenem first-line antibiotics.^14-17^ Hence, initial antibiotic choice in a febrile neutropenic patient who is critically ill presenting to the ICU will be carbapenems like Meropenem or Imipenem. The prevalence of carbapenem-resistant gram-negative organisms is alarming at present. According to the Indian Council of Medical Research (ICMR) data on non-neutropenic population, carbapenem resistance among *Enterobacteriaceae* is 35 to 50 %, *Pseudomonas spp* 47% and *Acinetobacter spp* 62%.^17^ Based on the epidemiology, current evidence and clinical experience the committee has identified risk factors for carbapenem resistance. Particular subgroups of patients, such as acute leukemia patients presenting to the ICU, a patient already on carbapenem shifted to ICU from the ward, previous antibiotic exposure in the last 1 month and patients on vasopressors are at risk of harboring carbapenem-resistant organisms.^18^ Hence in these groups of patients, initial empiric antibiotic regimen should include colistin/polymyxin B along with Imipenem or Meropenem (Table 3).

**Table 2:** Isolates from the blood of febrile neutropenic patients in Indian ICUs.

*Author, year*	*No of isolates*	*Gm negative isolates (%)*	*Common organisms (%)*	*Gram positive isolates (%)*	*Common organisms (%)*
Prabhash K^7^ 2010	484	68.1	*Pseudomonas* 30.4, *Acinetobacter* 11.6 *E coli* 10.9 *Klebsiella* 7.3 *Enterococcal spp*. 4.1	31.9	*Staph Aureus*- 12.6 (MRSA-2) CoNS- 10.5 *Streptococcus spp*. 4.6 *Burkholderia spp*. 2.9 *Enterobacter spp*. 2.3
Karanwal et al^8^ 2013	23	78	*E coli* 43, *Pseudomonas* 17.5	22	*Staph aureus* 22 CoNS- 4
Singh et al^9^ 2014	693	74.6	*E coli* 23.5, *Pseudomonas* 6.7	25.4	*Staph Aureus*- 34 (MRSA 13) *Enterococcus* 29
Rajendranath et al^10^ 2014	40	58.3	*E coli* 36.7, *Pseudomonas* 9.2	41.7	*Staph aureus*- 25 (MRSA- 2.5)
Sengar et al^11^ 2014	739	66	*E coli* 19, *Pseudomonas* 18.7 *Acinetobacter* 7.1 *Enterobacter* 4.8	34	CoNS-20 *Staph aureus*- 5.5 *Streptococcus* 3.9 *Enterococcus* 3.6
Lakshmaiah et al^12^	92	61.7	*E coli* 36	38.3	*Staph Aureus*- 36 (MRSA 9)
Vivek B et al^13^	285	63	*Pseudomonas* 22 *E coli* 21.4	37	CoNS 12.9 *Staph Aureus* 8

**Table 3:** Incidence of resistance among common gram-negative bacterial isolates in India (ICMR data)^17^

*Antibiotic*	*E Coli (%)*	*Klebsiella (%)*	*Entero-bacter (%)*	*Pseudo-monas (%)*	*Acineto-bacter (%)*
Amikacin	24	54	44	35	75
Aztreonam	-	-	-	48	87
Cefipime	79	80	80	41	81
Cefaperazone-Sulbactam	33	62	39	38	57
Cefotaxime	80	83	83	-	-
Ceftazidime	81	84	77	47	84
Ciprofloxacin	81	65	48	-	-
Colistin	1	1	0	10	22
Gentamicin	46	65	56	-	-
Imipenem	18	35	26	37	63
Levofloxacin	-	-	-	36	73
Meropenem	35	53	38	47	62
Netilmicin	12	42	18	45	69
Piperacillin-Tazobactam	43	68	57	46	83
Tetracyclin	64	42	16	-	55
Tobramycin	-	-	-	33	58
Trimethoprim-Sulpha-methoxazole	-	-	-	-	55

Vancomycin is not a standard part of empirical antibiotic therapy for the febrile neutropenic patient. In the western countries with predominant gram-positive bacteremia and high incidence of MRSA, studies have failed to show any benefit with empiric vancomycin in terms of fever or mortality.^19^ In India, with predominant gram-negative sepsis and low incidence of MRSA (35% as per ICMR data),^17^ Vancomycin/Teicoplanin is recommended as part of initial antibiotic regimen only in patients with suspected indwelling catheter infection (rigors following infusion, cellulitis at exit site), skin and soft tissue infection, severe mucositis, culture growing gram-positive cocci pending identification, previous MRSA colonization/infection and hemodynamic instability admitted from home/OPD.^7^

### Blood Culture Collection Procedure

### Evidence Statements

- We recommend a collection of at least two sets of blood cultures, with a set collected simultaneously from a peripheral site and one from the central line. In the case of a multi-lumen catheter, one set per lumen should be collected.
- Two blood culture sets from separate venepunctures should be sent if no central venous catheter is present.
- One set includes one aerobic and one anaerobic culture bottle. Blood culture volume should be at least 10 mL/bottle^7,20^ (I, A).

### Evidence Summary

The volume of blood is an important variable for detection of bloodstream infection volume of blood. Each ml of blood increased the yield (detection of positive culture) of blood cultures in adults by approximately 3%. Collection of two blood culture sets provide 80 to 90% yield of bloodstream pathogens in critically ill patients. In the pediatric population, smaller volumes of blood are suggested due to lesser total blood volume. The general consensus is not to exceed 1% of a patient's total blood volume.

### Empiric Antifungal Therapy in Febrile Neutropenic Patient

### Evidence Statements

Following patients should be considered for initiation of antifungal therapy when they present to ICU with shock or respiratory distress especially when they have a persistent or recurrent fever or clinical deterioration after > 3 days of broad-spectrum antibiotics (II, A)

- Allogenic hematopoietic stem cell transplantation (HSCT).
- Severe mucositis with diarrhea.
- Prolonged/anticipated duration of neutropenia >10 days.
- Worsening on broad-spectrum antibiotics like BL/BLI and Carbapenems.
- More than 3 weeks of steroids.
- History of invasive fungal infection.
- New onset lung infiltrates (since chest X-ray has low sensitivity, high-resolution computed tomography (HRCT) should be done in these patients).

- We recommend the use of caspofungin (echinocandin group)as initial antifungal therapy. Caspofungin should be avoided in patients with chronic liver disease (Child-Pugh C.)
- Anidulafungin and Micafungin can be considered if there are contraindications to use of caspofungin.
- Voriconazole is the drug of choice for proven, probable or possible aspergillosis. Due to its variable bio-availability, voriconazole should be administered IV. In patients with renal dysfunction, caspofungin can be given instead of IV voriconazole.
- Liposomal amphotericin B is the drug of choice for suspected or confirmed mucormycosis.
- All efforts should be made to confirm the presence of invasive fungal infection with the use of tests including CT chest, β–D-glucan, serum, and bronchoalveolar lavage (BAL) galactomannan, fungal culture. Tissue (lung/other clinically involved sites) biopsy should be performed if required, whenever feasible^21-25^ (II, A).
- We do not recommend the routine use of combination antifungal therapy for probable or proven invasive aspergillosis (III, A).

### Evidence Summary

Invasive *Candida* or *Aspergillus* infections have been demonstrated in the autopsy of patients who died of a neutropenic fever with no clinical evidence of invasive fungal infection (IFI) except for a continuous fever.^26^ It is estimated that approximately 15 to 45% of patients with prolonged neutropenia have an invasive fungal infection (IFI). IFI is difficult to diagnose both in critically ill patients and patients with febrile neutropenia. Invasive fungal infection is associated with high mortality in both these groups especially if treatment is delayed.

High-risk patients who have received intensive cytotoxic chemotherapy are at risk for invasive fungal infection. Yeast (primarily *Candida* species) and molds typically cause infections, which are manifested by persistent or recurrent fever in patients with prolonged neutropenia, rather than causing initial fever in the course of neutropenia.^27,28^ Empirical antifungal therapy is instituted for the treatment of “occult” fungal infection presenting as persistent neutropenic fever despite 4 to 7 days of empirical antibiotic therapy.^29^

The echinocandins have demonstrated significant fungicidal activity and treatment success against most of the *Candida* species in randomized clinical trials. Availability of intravenous formulation, limited drug interactions, favorable safety, and efficacy profile make them the first choice of empirical antifungal in critically ill patients including patients with febrile neutropenia. It is generally agreed upon that individual echinocandins namely caspofungin, micafungin and anidulafungin have similar efficacy and are interchangeable.^30^

### Recommended Dosage

- *Caspofungin*: loading dose of 70 mg followed by 50 mg daily. Dosage reduction is recommended for patients with moderate to severe hepatic dysfunction.
- *Micafungin*: 100 mg daily. No dose adjustment in the liver or renal failure.
- *Anidulafungin*: loading dose of 200 mg, followed by 100 mg daily. No dose adjustment is required in the liver or renal failure.

Limitations of echinocandin therapy should be kept in mind. Echinocandins have poor penetration in eye, CNS, and urine. Intuitively they should not be used for the treatment of fungal meningitis, endophthalmitis, and urinary tract infection. *Echinocandins* are not active against *Zygomycosis*.^31^

*Echinocandins* have activity against *Aspergillus* species. However, *Echinocandins* monotherapy as the first line for treatment of *Aspergillus* is not studied well. *Echinocandins* have shown to be effective in salvage therapy however they are not recommended as monotherapy for the primary treatment of IA due to lack of evidence.^29^

Lipid formulations of amphotericin B should be used as first-line treatment if *Mucormycosis* (Zygomycosis) is suspected. The recommended dose is 3 to 5 mg/kg daily.^32^

Invasive aspergillosis should be suspected in patients with persistent febrile neutropenia with the development of signs of pneumonia including lung infiltrate. Voriconazole can be used for suspected or proven cases of IPA. The dose of Voriconazole is 400 mg (6 mg/kg) twice daily for two doses, then mg/kg) twice daily. As mentioned above, Echinocandins are recommended for salvage therapy of aspergillosis.^33^

*Amphotericin* B deoxycholate should be avoided in patients with underlying renal impairment, patients on other nephrotoxic drugs such as cyclosporine or tacrolimus after allogeneic HSCT, or antibiotics, such as aminoglycosides and in patients with previous history of toxicity.

Recommended minimum duration of therapy for candidemia without metastatic complications is 2 weeks after documented clearance of *Candida* from the bloodstream, provided neutropenia and symptoms attributable to candidemia have resolved.^7^

Investigation for confirmation of invasive fungal infection:

Poor sensitivity of chest radiograph compared to CT scan for detection of pneumonia in this population should be kept in mind.^34^ The galactomannan assay is highly specific for *Aspergillus* species with some cross-reactivity with *Histoplasma capsulatum* and *Penicillium* species. The false positive reaction can occur with concomitant use of b-lactam/b-lactamase combinations, such as piperacillin-tazobactam.^22^

Marr et al have demonstrated 8.2% absolute reduction in mortality rates with the combination of voriconazole and anidulafungin in adult patients with hematologic malignancies (HMs) and hematopoietic stem cell transplantation (HSCT) having probable or proven Invasive aspergillosis (IA). However, this difference was not statistically significant.

Combination antifungal treatments are used with the rationale to maximize treatment by targeting multiple sites or metabolic pathways or different steps in the same pathway hence leading to an additive or synergistic effect. While *in vitro* studies on combination antifungals showed additive or synergistic effect; *in vivo* studies have given mixed results. While the combinations of azoles with echinocandins has shown additive or synergistic effects; combinations of polyenes with triazoles have demonstrated the antagonistic effect. Lack of *in vitro* findings has not been replicated with consistency. These *in vivo* studies are difficult to interpret due to the lack of validated protocols, variable test designs, and a definite end point. Combination antifungal therapy definitely increases the cost of treatment, has the potential to cause deleterious drug interactions, may add toxic effects and have the possibility of increased risk for resistance. In view of the lack of strong evidence in favor as well as potential harmful effects, routine use of a combination of combination antifungal therapy is not recommended.^35^

### Empiric Antiviral Therapy in Febrile Neutropenic Patient

### Evidence Statements

- There is no role of empirical antiviral therapy with febrile neutropenia (III, A)
- Acyclovir is recommended for the treatment of Varicella-Zoster virus (VZV) or Herpes Simplex virus (HSV) infection in the presence of clinical or laboratory evidence of infection is present (III, B)
- Serological (Immunoglobulin) tests should not be used to diagnose VZV or HSV infection (III, A)^36-39^
- Ganciclovir is recommended for the empiric therapy for Cytomegalovirus (CMV) in below-mentioned patients with high risk of CMV reactivation if they present with diarrhea/pneumonia not responding to antibiotics and antifungals (UPP, A)
  - Patients on T-cell-depleting agents such as fludarabine/purine analogs as well as rituximab
  - Patients on high dose steroids who develop diarrhea
  - Pneumonia not responding to antibiotics and antifungals

### Evidence Summary

General consensus does not recommend the empirical use of antiviral drugs in the management of febrile neutropenic patients except in cases with clinical or laboratory evidence of viral infection. Cellular immunity offered by T cells helps in controlling CMV replication preventing its recurrence hence patients receiving T-cell-depleting agents are at risk of CMV reactivation. No specific treatment for infections with RSV and parainfluenza viruses is recommended due to the lack of specific evidence.^40,41^

### Empiric Treatment Against Pneumocystis Carinii/Jiroveci Pneumonia (PCP) in febrile neutropenic patient

### Evidence Statements

- Treatment with sulfamethoxazole/trimethoprim should be considered in high-risk patients such as allogeneic HSCT, high-dose corticosteroid therapy administration of T-cell-depleting agents such as fludarabine/purine analogs and rituximab when such patients present with hypoxemic respiratory failure with or without radiological evidence of pneumocystis carinii pneumonia especially if they are not on PCP prophylaxis (III, A)
- Every attempt should be made to confirm PCP infection (I, A)

### Evidence Summary

Patients considered high risk for PCP infection are allogeneic HSCT recipients (Good evidence), autologous HSCT, high-dose corticosteroid therapy and patients receiving T-cell-depleting agents such as fludarabine, purine analogs and rituximab (moderate evidence).^42-44^ If they present with hypoxemic respiratory failure, then such patients should be on sulfamethoxazole/trimethoprim. Hypoxemia is the most characteristic abnormality in PCP pneumonia. Chest radiograph might be normal in early disease.

### Role for Empiric Antimicrobial Therapy for Tropical Infections like Malaria, Leptospirosis

### Evidence Statements

- There is no role for empirical antimicrobial therapy against tropical infections like malaria, leptospirosis (III, A)
- Documented tropical infections in neutropenic patients in ICU should be treated similarly as they are treated in non-neutropenic patients (UPP, B)

### Evidence Summary

There are occasional reports of malaria in patients on chemotherapy with a solid tumor and hematolymphoid malignancies with febrile neutropenia. In a series of 99 patients of acute leukemia on chemotherapy with febrile neutropenia, malaria was responsible for fever in only 4% of patients.^45^

Febrile neutropenia patient presenting to ICU often have thrombocytopenia due to the disease itself, chemotherapy or sepsis. Presence of fever and thrombocytopenia itself should not warrant empirical anti-malarial therapy even in a malaria-endemic country like India.

A high index of suspicion is warranted in a resident or traveler of malaria endemic area who presents with the classic triad of symptoms (fever, chills, and sweating). If malaria is suspected, peripheral smear for malaria parasite and rapid malaria antigen [e.g. Histidine-rich protein II (HRP-II) antigen of *Plasmodium falciparum* and common *Plasmodium* lactate dehydrogenase (pLDH) of *Plasmodium* species] should be performed early, and antimalarial therapy should be initiated in positive cases. With rapidity (diagnosis in less than an hour) and good negative predictive value of (98.2%) malaria antigen test, antimalarial therapy is restricted only to positive cases.^46^

There is lack of enough evidence documenting the etiological role of other tropical infections like Leptospirosis in a subset of patients with febrile neutropenia; hence we believe that until enough evidence is available, suspected or documented tropical infections in neutropenic patients in ICU should be treated similarly as they are treated in non-neutropenic patients.

### Role of Surveillance Cultures

### Evidence Statement

- We strongly recommend against repeated surveillance cultures as these do not help to guide antibiotic therapy (III, A)

### Evidence Summary

As most of the infections in neutropenic patients occur due to organisms in the respiratory or gastrointestinal tract, therefore surveillance culture seems to be a reasonable strategy in deciding the empiric antibiotic therapy in febrile neutropenia. The studies published in the 1980s and ’90s supported the practice of surveillance culture. However, there has been a very poor correlation between blood and fecal isolates in most of the studies.^47,48^

Widespread antimicrobial treatment may inhibit the growth or distort the proportion of different species found in fecal cultures. A recent study conducted in pediatric allogeneic HSCT patients has demonstrated a positive predictive value of 0.9% to bacterial surveillance cultures, with a sensitivity of 33.3% and a specificity of 47.4%. Surveillance cultures were not cost effective. The sampling and analyses require lots of laboratory and nursing resources.^49^ Another study in adults who got admitted for HSCT concluded that surveillance blood cultures in patients who have undergone HSCT do not identify bloodstream infections. The number of positive blood cultures was not helpful in determining which patients had an infection.^50^

### Role of Source Control in the Treatment of a Febrile Neutropenic Patient

### Evidence Statements

- We recommend that in patients with febrile neutropenia with a clinically documented source of infection; immediate intervention should be undertaken for source control (III, A)
- We recommend that Non-tunnelled (short term) central venous catheter (CVC) should be promptly removed in following cases (III, A).^52^
  - CVC is an obvious source of infection.
  - Patient not improving/deteriorating in spite of 24 hours of antibiotics and resuscitation with no other source of infection evident
- We recommend that long term central venous catheter should be promptly removed in presence of any of the following (III, A).^52^
  - Documented line/blood infection with S. aureus, or Candida species
  - Tunnel infection or port abscess
  - Septic thrombosis, endocarditis or osteomyelitis
- We suggest considering the removal of long term central venous catheter in the absence of above-mentioned features if there is deterioration or no improvement in patients condition and no other obvious source of infection is evident (UPP, B)

### Evidence Summary

Control of source in the form of drainage of an abscess, debridement of infected necrotic tissue and removal of a potentially infected device is of paramount importance. Foci of infection readily amenable to source control include but not limited to intra-abdominal abscesses, gastrointestinal perforation, ischaemic bowel or volvulus, cholangitis, cholecystitis, pyelonephritis associated with obstruction or abscess, necrotizing soft tissue infection, empyema, septic arthritis, and implanted device infections. There is general agreement that source control should be done at the earliest to reduce microbiological burden, and mere antibiotics and resuscitation would not achieve cure unless adequate source control is done. If Vascular catheters are suspected, its prompt removal should be considered. It is important to note that the classical clinical signs of infection (rubor, calor, dolor, etc) be absent due to low neutrophil counts.^51^

Patients with *S. aureus* catheter-related bloodstream infection (CRBSI) have a significantly higher risk of hematogenous complications with the retained foreign body, especially in immunocompromised patients. Retention of the catheter has shown to worsen the outcome of candidaemia.^52^

### Antibiotics De-escalation

### Recommendations

- Antimicrobial de-escalation should be implemented in the following situations: (III, A)
  - Once and if a pathogen is identified, we recommend de-escalation to an antibiotic that the organism is susceptible to.
  - We recommend treating with appropriate agents based on the site and pathogen until the patient is afebrile for at least 48 hours and there is evidence of marrow recovery (neutrophil count ≥ 500 cells/mm^3^).
  - In patients without microbiologically documented infection we recommend continuing empirical antimicrobials until the patient is afebrile for at least 48 hours and there is evidence of marrow recovery (neutrophil count ≥ 500 cells/mm^3^)

### Evidence Summary

Data on de-escalation strategies in neutropenic patients after identification of a clinically relevant pathogen is scant, and there is no data on de-escalation when no pathogen has been identified. Although antibiotics are required to treat an occult infection during neutropenia, marrow recovery is necessary to protect the patient.^18^

### Antibiotics for Multidrug-resistant Bacteria

### Useful Practice Points

Antibiotics like Fosfomycin, tigecycline and minocycline may be considered in infection with multidrug-resistant bacteria in the presence of *in vitro* susceptibility after considering the *in vivo* penetration at the source of sepsis.

### Evidence Summary

**Table:** 

	*Fosfomycin*	*Tigecycline*	*Minocycline*
Good activity	*E. coli, Citrobacter sp, Proteus mirabilis, Streptococcus sp, Staphylococcus aureus* (MSSA and MRSA) and *Enterococcus faecalis*	Most *Entero-bacteriaceae, Acinetobacter sp, Staphy-lococcus aureus* (MSSA, MRSA), *Enterococcus sp* (VRE) and anaerobes	Most *Entero-bacteriaceae, Acinetobacter sp, Staphylococcus aureus* (MSSA, MRSA), *Enterococcus sp* (VRE) and *anaerobes*
Poor activity	*Pseudomonas sp, Acinetobacter sp, Bacteroidesspp* (*Anaerobes*), *Coagulase negative staphylococcus, Morganella-morganii* and *Enterococcus faecium*	*Pseudomonas sp, Proteus sp, Providencia sp* and *Morganella sp.*	*Pseudomonas sp, Proteus sp, Providencia sp* and *Morganella sp.*
Uses	Bacteraemia, VAP, UTI, wound infection and meningitis	Skin and soft tissue infection and intra-abdominal infection	Ventilator - associated pneumonia (VAP) urinary tract infections (UTI) Hospital/healthcare -Acquired Pneumonia (HAP)
Caution	High sodium load	Should not be used as a sole agent in Bacteriamia, pnuemonia	

VAP: Ventilator Associated Pneumonia

HAP: Hospital/healthcare Acquired Pneumonia

Urinary Tract Infections (UTI)
