# Supplementary material for: Indian Antimicrobial Prescription Guidelines in Critically Ill Immunocompromised Patients
Source: Indian J Crit Care Med. 2019 Jan;23(Suppl 1):S64–96. doi: 10.5005/jp-journals-10071-23102 (PMC6734470; doi:10.5005/jp-journals-10071-23102)
Supplement: Related texts

# Article commentary: PART 2. SOLID ORGAN TRANSPLANT (SOT) RECIPIENT IN THE INTENSIVE CARE UNIT (ICU)

Infectious complications in solid organ transplant (SOT) recipients pose unique challenges when such patients require intensive care unit (ICU) care. The net immunological state of these patients is a measure of an individual's unique susceptibility to infection and incorporates an assessment of several important contributing factors.

- Pretransplant diagnosis or treatment
- Nature of organ transplant received (e.g., lung vs. liver organ transplant)
- Dose, duration, and choice of maintenance immunosuppression
- Comorbidities (e.g., viral coinfection [hepatitis C virus (HCV), cytomegalovirus (CMV)], malnutrition, end-organ failure [cirrhosis, chronic kidney disease])
- Breaches of the mucocutaneous barrier: Indwelling devices, mucositis

Detection of infection in SOT recipients is difficult and requires a high index of suspicion, as fever and localizing signs are usually absent in the majority of the patients. Detection of infections should include an assessment of risk factors, detailed history, and physical examination. The infections in SOT patients can be categorized as follows.

- C–Community-acquired
- R–Reactivation
- E–Epidemiologic exposure
- D–Donor-derived
- I–Iatrogenic
- T–Travel related

It is advisable to have a syndrome-based approach (e.g., Nonspecific febrile illness, pneumonia, urinary tract, central nervous system) at first and then narrow the differential diagnoses of possible organisms that could cause the clinical presentation(s).

Microbiological diagnosis is crucial in this patient group. In the context of extensive differential diagnoses, the value of early and specific diagnostics with the use of invasive procedures if necessary (bronchoscopy, tissue biopsy, or aspiration of collections) to obtain specimens cannot be underestimated. After transplantation, serologic techniques are of limited use because transplant recipients may not mount timely serologic responses. Thus, antigen detection or molecular nucleic acid detection assays are preferred over serologic testing.

**Table 1:** Timeline of infections after a solid organ transplant

*Infection characteristics*	*Timeline*		
	*0-1-month Post-SOT*	*1 to 6-months Post-SOT*	*More than 6-months Post-SOT*
Infection Types	Nosocomial infection Pneumonia-HAP, VAP, CRBSI, CAUTI, Post-surgical Infections Donor-derived infections	Opportunistic Infections, Reactivation of latent infections	Usually community-acquired infection Reactivation of latent infections during immunosuppression
Bacterial	Resistant Bacteria MRSA VRE MDR gram-negative bacilli C. difficile associated infections	M. tuberculosis Listeria Legionella Nocardia	Ongoing risk of M. tuberculosis, Listeria, Legionella, Nocardia if immunosuppression continued Graft-related infections UTI in Renal transplant Pneumonia in lung transplant Cholangitis in liver transplant Community-acquired pneumonia
Viral	HSV (in absence of anti-HSV prophylaxis) HIV West Nile virus	BK Virus, Adenovirus RSV HCV Reactivation CMV, EBV, HSV, VZV	Recurrent HSV, VZV Adenovirus RSV HCV Reactivation Late-onset CMV, EBV related PTLD
Fungal	Candida (likely to be Fluconazole resistant) Early Aspergillosis (Uncommon)	*Aspergillus* *Cryptococcus* *Zygomycetes* PCP if not on prophylaxis	During intense immunosuppression: Aspergillus Cryptococcus Zygomycetes PCP
Parasitic	Rare	Toxoplasma Strongyloides Leishmania, Trypanosoma	Ongoing risk of Toxoplasma Strongyloides Leishmania, Trypanosoma if immunosuppression intensified

The timeline of post-transplant infections reflects the post-transplantation relationship between the recipient's epidemiologic exposures and immunosuppressive strategy employed (Table 1). It is used to establish a differential diagnosis for infectious syndromes at various stages after transplantation. Infections occurring outside the usual period or of unusual severity suggest excessive immunosuppression or epidemiologic hazard. Most centers use a variation of standard ‘triple immunosuppression’ (prednisone, calcineurin inhibitor, antimetabolite such as mycophenolate mofetil).

## Evidence Statements

- Infections in the first month (0–30 days) of post SOT period should be treated similarly to the treatment of a non-immunocompromised postoperative patient (I, A).

Approach to initial diagnostic workup and empiric therapy in post SOT recipients (30–180 days).

- We recommend early BAL in patients with suspected pneumonia coming to ICU (I, A). We recommend that the BAL fluid should be tested for (I, A).

Total and differential cell counts

Microbiology

□ *Cultures*: Aerobic culture for bacteria, mycobacterial growth indicator tube (MGIT) for mycobacterium tuberculosis, fungal culture
□ Stains and immunohistochemistry

*Gram stain*: Bacterial

*KOH preparation/Calcofluor white*: Fungal

*Auramine-rhodamine, auramine-o, or ziehl-neelson*: Mycobacterial

*Modified acid-fast stain (kinyoun)*: Nocardia

Silver methenamine: Pneumocystis carinii pneumonia, fungal

□ *Galactomannan assay*: Negative predictive value < 0.5, positive predictive value > 3
□ Polymerase chain reaction (PCR)

*Mycobacterium tuberculosis*: Cartridge Based Nucleic Acid Amplification Test (CB-NAAT or GeneXpert)

Multiplex PCR assay

□ Quantitative or semiquantitative cultures particularly for pneumonia.

Following organisms are diagnostic of infections. If organisms are identified it is less likely to be the contaminants/colonizers and should be treated.

  - Pneumocystis carinii
  - Toxoplasma gondii
  - Strongyloides stercoralis
  - Legionella pneumophila
  - Cryptococcus neoformans
  - Histoplasma capsulatum
  - Mycobacterium tuberculosis
  - Mycoplasma pneumoniae
  - Influenza a and b viruses
  - Respiratory syncytial virus
  - Cytology

- We recommend HRCT chest for the diagnosis of nodular infiltrates to rule out invasive aspergillosis (I, A).
- We recommend empiric management with carbapenem and azithromycin till causative organisms are identified (I, A).
- We recommend adding polymyxins if the patient is admitted with≥ 48 hours before symptoms (I, A)

Medical care (hemodialysis, wound care, immunosuppressants) within the previous 30 days (UPP, B)

Hospitalization in an acute care hospital ≥ 2 days within the prior 90 days (UPP, B)

- We recommend voriconazole in the dose of 6 mg/kg bd for 1-day f/b 3mg/kg bd (I, A)
- As a salvage therapy, we recommend Caspofungin (dose 70 mg IV followed by 50 mg iv daily) (I, A)
- We recommend therapeutic drug level monitoring of the voriconazole when using these agents for the treatment of Invasive Aspergillosis (I, A).

## Evidence Summary

During the first month after SOT, opportunistic infections are generally absent as the full effect of immunosuppression has not yet been established. In this period donor-derived or recipient-derived viremia, candidemia or technical complications related to surgery are common.^1^ Most infections are caused by gram-negative bacterial (GNB). Bacterial infections are either nosocomial or healthcare-associated (27.4% and 49.8% respectively); the remaining 22.9% are community-acquired.^2^ The urinary tract infection (UTI) is the most common primary source of GNB infections in 55.2% of SOT recipients. Gastrointestinal infections are the second most common infections (15.2%). Other infections include infections of the respiratory tract (3.6%), intravascular catheters (3.6%) and skin and soft tissue (2.7%) are also seen in SOT recipients in decreasing order of occurrence. E. coli has been found to be the most common GNB (36.8%), followed by *K. pneumoniae* (14.3%), *P. aeruginosa* (13.0%), *Enterobacter cloacae* (4.9%) and *Citrobacter freundii* (4.5%) whereas polymicrobial infections occurred in 8.1% of cases. The incidence of GNB infections shows a declining trend to fall to 25.7 (95% CI: 20.1–32.1)from 2 to 12 months. It further declines to 8.2 (95% CI: 6.7– 10.0) after 12 months per 1000 person-years following SOT.^1,2^

**Table 2:** Common infections in SOT recipients in the period 30 to 180 days 1–41

*Bacterial infections*		*Viral infections*	*Mycobacterial infections*	*Fungal infections*
Community-acquired pathogens: *S pneumoniae,* *H influenzae,* *M catarrhalis,* *S aureus,* *Mycoplasma pneumoniae,* *Chlamydia pneumoniae* *Nocardia rhodococus*	Nosocomial pathogens Gram-negative bacilli (*E. coli, P. aeruginosa, K. pneumoniae*), MRSA, *Legionella* *Clostridiumdifficile*	*Respiratory viruses*: Influenza, Parainfluenza, RSV, Human metapneumovirus, Adenovirus, Coronavirus, entero/Rhinovirus CMV, Herpes simplex virus (HSV), Varicella-zoster virus (VZV)	Tuberculosis Nontuberculous mycobacteria: mycobacterium avium complex (MAC), M. abscessus	Invasive moulds: *Aspergillus, Zygomycetes, Fusarium, Scedosporium* *P jirovecii* pneumonia (PJP/PCP) *Cryptococcus* (neoformans) *Endemic mycoses*: Histoplasmosis, Coccidiomycosis, Blastomycosis

Indian data shows that UTI is the most common infection.^3^ In 169 renal allograft recipients, they observed that 23.6% of the SOT recipients develop UTI during first 4 weeks and E. coli is the most common causative agent (12.6%). CMV is the most common (prevalence of CMV 21.8%) between 4 weeks to 3 months after renal transplantation and can cause allograft loss. Tuberculosis reactivated commonly between 3 months to 1-year post-transplant (prevalence of tuberculosis being 10.6%). *Pneumocystis carinii* and *Aspergillus* are serious hazards which usually occur after 1 year. Aspergillus infection is the leading fungal infection (10.0%).^3^ Another retrospective study in of 144 renal transplant patients also found that UTI was the most common infection (34.5%), followed by viral (31.2%), sepsis (15.2%), mycobacterial (9.7%) and fungal (6.2%). Parasitic infections (giardiasis and Strongyloides hyper infection syndrome) occurred in 2 (1.4%) patients. CMV accounted for 14.5% and BK virus (*Polyomavirus hominis1)* for 5.5% of total infections.^4^ One prospective observational study in 45 renal transplant recipients reported a similar finding. In the same study, other infections like tuberculosis (*n* = 8), CMV (*n* = 6), candidiasis (*n* = 7), hepatitis (*n* = 11), cryptosporidiosis and pneumocystis (n = 10) and in 7 patients simultaneous infections with two organisms were seen. *E. coli* (33.3%) was the most common organism grown in culture followed by *Klebsiella* (20%), *Acinetobacter* (20%), Proteus (6.67%), and others such as *Staphylococcus* or *Pseudomonas* (6.67%). The incidence of MDR was found to be 31.8%.^5^

A study on bacterial infections in SOT recipients in liver transplant recipients observed that overall incidence of infections during the first-month post-transplant was 80.2% and the incidence of bacterial pneumonia in the first six months was 21.3% (101/475). A prospective study of 475 liver transplant recipients found that the frequent pneumonia isolates were *Enterobacteriaceae, Acinetobacter baumannii, P. aeruginosa* and *Staphylococcus aureus*. Gram-negative *bacilli* accounted for 69.6% of all pneumonia pathogens out of which MDR rate was 58.9%.^6^

One prospective multicenter study involving 35 centers reported the incidence of pneumonia after SOT as 10.1 episodes/1000 recipients/year and bacteria (87.1%), virus (29%), and fungi (6.4%) were the common causative agents. A multidrug-resistant bacterium is isolated in 18.2%, 40%, and 100% of patients with CAP, HCAP, and HAP (p = 0.007), respectively. Overall, 11.1% of patients admitted to the intensive care unit, 3.7% developed graft rejection, and graft function deteriorated in 18.5%. In-hospital mortality was 1.9 %.^7^ In renal transplant recipients, bacterial infections are the most common cause of pneumonia. A retrospective study of 406 renal transplant recipients observed that incidence of healthcare-associated pneumonia is 56% and bacterial infections are the most common cause (31% of the patients), especially *haemophilus influenzae, stenotrophomonas maltophilia,* and pseudomonas *aeruginosa.*^8^ Another prospective study of 610 kidney transplant recipients also observed 60 episodes of pneumonia in 54 patients (8.8%), of which 23 had a nosocomial origin (38%) and 37 were community-acquired infections(62%). Bacterial infection is the most frequent etiology (44%), followed by fungal in 4 (7%) and viral in 2 (3.5%). The most commonly isolated microorganism in nosocomial pneumonia is *P. aeruginosa* (26%, among which 50% were multidrug resistant). In 34% episodes, no microorganism is isolated. The most common pathogen among community-acquired pneumonias is *S. pneumoniae* (11%). In 54% of cases, there is no microbiologic confirmation of disease. The overall accuracy of bronchoalveolar lavage is 72%. When 21 patients with pneumonia (35%) were admitted to the ICU; of these, 14 had a nosocomial infection (60%) and 9 (15%) died due to the infection (p = 0.001).^9^

Data on heart transplant recipients has also shown that pneumonia is the most common complication. A prospective review of 307 heart transplant recipients found 21.1 cases of pneumonia per 100 heart transplantations. 75% of the cases occurred in the first 3 months, 82 causal agents are identified, of which 60% were opportunistic, 25% are nosocomial, and 15% were community-acquired. The most frequent isolates were CMV,20 aspergillus species,^13^ and pneumocystis carinii.^11^ Hemoptysis occurred more frequently in aspergillus pneumonias than in other pneumonias (54% *vs*. 6%, respectively; p < .05); aspergillus pneumonia is the only type of pneumonia during which cavitated nodules were noted on thoracic radiographs. The overall mortality rate was found to be 30.8%.^10^ Lenner et al reported 47 of 159 heart transplant recipients (29.9%) had 81 pulmonary complications. 1including Pneumonia (n = 27), and bronchitis (n = 15).^11^ A retrospective review of 34 heart transplant recipients (31.3%) who developed pulmonary complications, within first 6 months post-transplant showed Hospital-acquired bacterial pneumonia in 5 patients, fungal pneumonia in 3 patients, a post-transplant lymphoproliferative disease in 1 patient, and community-acquired pneumonia in 1 patient. Pneumonia-related mortality rate was 14.7% due to early-onset nosocomial pneumonias where bacterial and opportunistic microorganisms organisms were more commonly seen.^12^

SOT recipients are at risk of developing bacterial infections like nocardia.^13^ The risk of developing nocardiosis after SOT varies with the type of organ transplanted, e.g. lung transplant. In a review of 5126 organ transplant recipients has demonstrated that highest nocardial infection rate was seen among lung transplant recipients (3.5%).^14^

Due to frequent exposure to antibiotics and repeated hospitalizations; SOT patients are at risk of developing intra-abdominal infections (IAI). IAI is commonly seen as a complication in post-liver, pancreas, multi-visceral transplants. Superinfection with MDR pathogens occurs frequently causing tertiary peritonitis.^15^ Clostridium difficile-associated disease (CDAD) is the most common cause of nosocomial diarrhea. One cohort study involving 4472 SOT patients observed that 42 episodes of CDAD were diagnosed in 36 patients (0.94%). Median onset of infection was 31.5 days (range 6–741). It occurred during the first month after transplantation in half the cases and overall the prognosis was good.^16^

SOT recipients are at risk to develop viral infections leading to various nonspecific viral syndromes. Common viruses seen are CMV, EBV, and other viruses like herpesvirus,^8^ Zika virus, RNA respiratory viruses, adenovirus, norovirus, and polyomaviruses.^17^ Among viruses CMV virus disease which occurs during the first 3 months. With the introduction of CMV prophylaxis, this onset has been delayed. The seroprevalence rate of CMV ranges from 30–97%.^18^

The incidence of PCP in SOT recipients is variable. In a retrospective study of 1192 renal transplant patients, it was reported to be 0.6 to 9%. They obsereved that the incidence of PCP with a moderate Cyclosporine based immunosuppressive regimen is low and seems to occur only in cases of additional immunosuppressive cofactors.^19^ In another retrospevtive study of 601 renal transplant recipients, PCP incidence was 2.2%.^20^ In liver transplant recipients (154 adult patients) PCP occurred in 5.2% and the authors observed that patients who developed PCP had more episodes of rejection (p < 0.05), received more OKT3 (p < 0.05), a prednisone (p < 0.05) than controls.^21^ Another retrospective study of 43 adult OLT recipients showed that the incidence of PCP was 0.9%. Most of the patients developed PCP at around 1 year of post-OLT, and the risk of PCP was closely related to strong immunosuppressive regimen. Thus they advised that routine PCP prophylaxis for 12 months be continued for 12 months, among patients receiving antirejection treatment.^22^

Invasive fungal infection (IFIs) frequently complicates post SOT course. Cryptococcosis is a significant opportunistic infection in SOT recipients following aspergillosis and candidiasis. CSF analysis is highly recommended to diagnose underlying CNS disease in suspected cases.^23^ In Lung and heart transplant, – 2% incidence IFI of lung was noted and even dissemination can occur. *Cryptococcus* can colonize the airways of lung transplant recipients and can cause endobronchial fungal infection. It can present with skin manifestations and Immune reconstitution syndrome as well.^24,25^ In heart transplant recipients incidence of *cryptococcus* is 3%, it manifests as sepsis and is associated with high mortality.^26,27^ In renal transplant recipients incidence of *cryptococcus* is 2.8% manifests as cryptoccocal necrotizing soft tissue infection.^28^ In liver transplant recipients the incidence is 2.4 % and liver failure is independently associated with Cryptoccocal meningitis mortality.^29^ However, with wider employment of antifungal prophylaxis and improvements in transplantation practices, there is a decline in the overall incidence of IFIs.^30,31^ SOT reciepnts have risk of developing aspergillous particulary in lung trsnplant. A retrospective study involving 362 lung transplant recipients found that 105/335 (31%) patients had evidence of aspergillus infection (colonisation or invasion), 83 (25%) patients had colonisation and 22 (6%) patients had radiographic or histological evidence of invasive disease. Most of the infections occurred within the first 3 months after transplantation. Invasive aspergillosis (IA) was associated with 58% mortality after 2 years, while colonisation was associated with increased mortality after 5 years compared non-colonised patients (p < 0.05).^32^ Hambrecht et alcompared voriconazole with amphotericin b in their large randomized trial for the treatment of invasive aspergillosis (IA) in immunocompromised patients.^33^ After this trial voriconazole had been considered the preferred agent for IA. In their study they found that at week 12, there were more successful outcomes 52.8% patients in the voriconazole group (complete response 20.8% and partial response in 31.9%) compared to 31.6% in the amphotericin B group (complete response 16.5% partial response in 15%). The survival rate was better at 12 weeks in voriconazole compared to amphotericin B group. (71% vs. 58%) (HR- 0.59; 95% CI- 0.40 to 0.88). After this study several studies were conducted for use of voriconazole for the treatment of IA, especially in patients of solid organ transplant recipient. J. Fortun et al in their case series reported of 100 % complete response in four patients (two liver transplant, one lung transplant and one kidney transplant) of IA after treatment with voriconazole.^34^ Denning et al in their study showed good response in IA treated with voriconazole; 56 out of 60 patients in voriconazole group were treated successfully.^35^ Veroux et al reported complete response in four kidney transplant patients with IA treated with voriconazole.^36^ In another report that included 11 SOT recipients with central nervous system aspergillosis treated with voriconazole, the favorable response rate was 36% and survival was 31%.^37^ Voriconazole was successfully used in heart transplant recipients as first-line and salvage therapy for IA.^38,39^ Plasma drug level monitoring is important when voriconazole is used as the plasma levels achieved are variable and very often do not reach therapeutic levels in the plasma, requiring dose adjustments.^40^ The fact that clinical efficacy is dependent on the achievement of therapeutic drug levels has been well established.^41^

## CMV management

- We recommend IV ganciclovir 5mg/kg twice a day as the initial treatment for (I, A):
  - Severe or life-threatening CMV disease
  - Patients with high and increasing viral load
  - Patients with questionable gastrointestinal absorption
- Oral valganciclovir 900 mg once a day is an effective initial therapy for mild to moderate CMV disease (I, A), or as a step down to iv ganciclovir after clinical improvement (II, B)
- We recommend against the use of acyclovir and oral ganciclovir for treating CMV disease (UPP, A).
- IV gamma globulin or preferably CMV-specific gamma globulin if available in the dose of 1 gm/kg over two days may be considered for patients with life-threatening disease, CMV pneumonitis (II, B).
- We recommend a duration of treatment for a minimum of two weeks. It should be continued until viral eradication is achieved either by weekly monitoring for viral load by real-time quantitative PCR (I, A).
- Therapy should be extended beyond two weeks if the clinical resolution is not seen or virus load continues to be high (I, A).
- After completion of full-dose antiviral treatment, a 1 to 3 months course of secondary prophylaxis may be considered depending on the clinical situation (II, B).
- Cautious reduction in immunosuppression should be considered in SOT patients presenting with CMV disease, especially if the disease is moderate to severe (II, B).

## Evidence Summary

Drugs used for the treatment of CMV disease are IV Ganciclovir and oral valganciclovir. Oral ganciclovir should not be used due to poor bioavailability. Ganciclovir through IV route has been demonstrated as the treatment of choice in many trials.^42-44^ Asberg et al in a randomized controlled trial compared the outcome of CMV disease after treatment with IV Ganciclovir and oral valganciclovir. Three hundred twenty-one SOT recipients were enrolled and randomized to receive either twice daily intravenous ganciclovir or oral valganciclovir for 21 days followed by once daily valganciclovir until day 49 in all the patients. All patients were followed up for 1 year. The success rate was the same in both the groups with a similar rate of clinical and viral eradication. The clinical recurrence rate was also not statistically different in both the groups.^43^ In a retrospective study, the response to therapy was assessed using RT-PCR (2262 samples) and antigenemia using pp65 assay (1285 specimens). Both methods had > 90% specificity, but RT-PCR had better sensitivity. The authors concluded that RT-PCR was a more reliable tool to monitor the response to therapy.^45^ Failure to eradicate DNA-emia was the only independent predictor of recurrence in both the groups. The efficacy for the eradication of viremia was similar in both the groups, still many patients were viraemic even after treatment for 21 days, hence, duration of treatment should be individualized and based on clinical resolution and virologic clearance.^46-48^ There is a direct association between viral suppression below the lower limit of quantified test and disease resolution. Rapid resolution of CMV disease is seen with lower pre-treatment viral load (lower than 18,200 IU/mL).^49^

## PCP Management

- We recommend trimethoprim-sulfamethoxazole (TMP-SMX) as the first-line agent and drug of choice with the Trimethoprim component being 20 mg/kg/day in 3 to 4 divided doses (I, A).
- In severe infections, if available, intravenous pentamidine probably remains the second-line agent after TMP-SMX (II, A).
- In patients with hypoxemia (PaO_2_ < 70 mmHg on room air), adjunctive corticosteroids should be administered with antimicrobial therapy, ideally within 72 hours of initiating antimicrobial therapy for maximum benefit (II, A). The dose of steroids should be 1 mg/kg/day prednisone (or equivalent) given in two divided doses daily for 5 to 7 days (II, A). Steroids should be tapered over a period of 7 to 14 days (II, B).
- Duration of antimicrobial therapy should be for at least 14 days (I, B).

## Evidence Summary

TMP-SMX acts by interfering with folate metabolism and remains the drug of choice for treatment of PCP in SOT patients, HIV patients, and non-HIV patients. TMP-SMX has high efficacy and availability in both oral and IV preparation with good oral bioavailability too.^50^ Intravenous pentamidine has been found to be equally effective in HIV-infected patients and remains the second line of choice for treatment of PCP in SOT patients.^51-55^ However, the use of pentamidine has been largely limited in view of its numerous toxicities in 71% patients leading to withdrawal in around 18 % patients.^52^ The optimal duration of therapy is usually 14 days which can be extended to 21 days in severe cases with slow clinical improvement.^56^ Adjunctive glucocorticoids are recommended for HIV-positive patients with moderate to severe PCP, defined as PaO_2_ < 70 mmHg while breathing ambient room air.^57^ The benefit in survival from corticosteroids begins during the first 72 hours of treatment.^58^ Bolée et al's study found a trend for longer survival in patients who received adjunctive steroids (p = 0.07).^59^ In Pareja et al's study, there was no difference in the mortality rates of patients treated with adjunctive high-dose steroids, but they did spend less time on mechanical ventilation compared to patients not managed with steroids.^60^

## Tuberculosis in SOT Recipient

## Evidence Statements

- The diagnosis of active TB in transplant recipients requires a high index of suspicion. Although the diagnostic modalities and treatment of TB in SOT patients remains the same as that in immunocompetent hosts, these individuals often require an invasive procedure, such as bronchoscopy with bronchoalveolar lavage or lung biopsy (I, A).
- Rifamycins, particularly rifampin, reduce serum concentrations of tacrolimus, cyclosporine, rapamycin (sirolimus), and everolimus via induction of the cytochrome p450 isoenzyme CYP3A4, necessary dose adjustments, and therapeutic drug monitoring are warranted to avoid development of rejection (II, A). When rifampin is not used, a longer than usual duration of treatment is required (II, B).

## Evidence Summary

Given that tuberculosis is an immunological disease and with the high prevalence of TB in India, the incidence of active tuberculosis infection is higher among SOT recipients as compared to the general population.^61,62^ The diagnosis of TB in SOT recipients presents challenges that may lead to treatment delay. These include atypical clinical presentations, increased likelihood of negative tuberculin skin tests and/or IGRA, and negative sputum smear results despite active disease makes TB diagnosis in SOT recipients a challenge.^61,63-65^ One-third to one-half of cases of tuberculosis after transplant are disseminated or extrapulmonary. Lung transplant recipients are most likely to develop pulmonary manifestations of TB. Similarly, drug-drug interactions between immunosuppressive and AKT, allograft-related drug toxicities, and inadequate immune responses to TB makes treatment of TB in transplant recipients also very challenging.^63-65^

## Infective diarrhea in SOT patients

## Evidence Statements

- We recommend empiric management of GI infections with ceftriaxone iv + ganciclovir 5g/kg BD IV and vancomycin 125 mg PO QID (if the patient is already on antibiotics to cover CDI) till definitive diagnosis is made (I, A).
- If the patient is in septic shock, based on local resistance pattern, and previous drug history of patient consider carbapenems (UPP, A).
- We recommend cessation of the inciting antimicrobial agent whenever possible (II, A).
- We recommend NAAT for the diagnosis of CDI (I, A).
- For mild-to-moderate CDI we recommend oral metronidazole (I, A). Dose of metronidazole 500 mg TID for adults.
- We recommend oral vancomycin for the treatment of severe CDI (IA). The accepted dose of vancomycin is 125 mg qid for adults and 40 to 50 mg/kg/day divided QID for pediatric patients (not to exceed adult dosing).
- In severe CDI with complications, a dose of oral vancomycin may be increased up to 500 mg orally QID.
- Vancomycin may be administered by retention enema (IIB), and intravenous metronidazole may be added (IIC).
- Feacal transplant may be considered in recurrent or relapsing CDI (II, B).
- We suggest consideration for surgical intervention in cases of complicated CDI. (II, B).
- In cases of multiple recurrences of CDI, we recommend prolonged courses of oral vancomycin, either in a tapering or pulse dose schedule (II, A). Fidaxomicin can be used if available (II, B)

**Table 3:** Common causes of diarrhea in SOT patients

*Common* CMV infection Clostridium difficile infections (CDI) Small bowel bacterial growth (SBBO): *Escherichia coli, Campylobacter, Shigella, Salmonella* MMF drug therapy *Unusual* Infections other than CMV and bacterial: Viruses (adenovirus, astrovirus, human herpes virus 6/hhv-6, norovirus, dengue fever), fungi (*candida*), and parasites *(blastocystis, hymenolepis nana, trichuristrichiura, isospora belli,* and *Entamoeba* Non-infectious colitis (IBD) Drugs other than MMF Rare GVHD PTLD Microscopic colitis Colon cancer

## Evidence Summary

Due to frequent exposure to antibiotics and repeated hospitalizations SOT patients are a risk of developing intra-abdominal infections (IAI). IAI is commonly seen as a complication in post-liver, pancreas, multivisceral transplantation and superinfection with MDR pathogens occur frequently causing tertiary peritonitis.^66^ Common causes of diarrhea are as mentioned in Table 3. The treatment of choice for CDI had been oral vancomycin. Vancomycin has been shown to have much better efficacy compared to metronidazole in many studies. Zar et al in their randomized controlled trial in 150 patients found that both metronidazole and vancomycin were equally effective in treating mild CDI, but Vancomycin was much more effective in treating severe CDI.^67^ The clinical cure rate in mild CDI was 90% and 98% with metronidazole and vancomycin respectively (p = 0.36). In severe CDI the cure rates were 76% and 97% respectively (p = 0.02). Recurrence rates were also comparable in both the groups. Fekety et al in their randomized controlled trial compared two doses regimen of vancomycin in 46 patients viz. 125 mg four times a day *vs*. 500 mg four times a day.^68^ They found no difference in measurable responses to the two regimens. Since 125 mg four times a day is more cost-effective 125 mg dose is recommended. Even 125 mg-dose produces stool concentration of vancomycin of around 100 times more than the minimal inhibitory concentration (MIC) for C. difficile.^69^ The usual dose of oral vancomycin for children is 40mg/kg daily given in three or four divided doses. Fecal microbiota transplantation (FMT) in the management of refractory CDI has gained popularity recently, although in SOT recipients it has theoretical safety concerns. A series of cases (75 adults and 5 pediatric patients) treated with FMT for recurrent, refractory, and severe and/or overlap of recurrent/refractory and severe CDI had 78% cure rate after first FMT.^70^ There were no related infectious complications or adverse events in these high-risk patients.

## CNS Infections

## Evidence Statements

- We recommend initial workup for suspected CNS infections should include (I, A)
  - MRI over CT scan
  - CSF analysis including India ink preparation
  - Rapid multiplex PCR on CSF
  - Serum cryptococcal antigen
- We recommend empiric treatment to be started with Ceftriaxone + Vancomycin + Acyclovir (I, A)
- We recommend Liposomal Amphotericin B as the initial treatment for *Cryptococcus*. (I, A)

## Evidence Summary

SOT patients with altered sensorium should be evaluated with detailed workup. Multifactorial etiologies coexist which are often obscured in these group of patients.^71^ Common pathogens causing CNS infections in SOT recipients are mentioned in Table 4. Although each imaging modality has unique insight to diagnose pathophysiology, but magnetic resonance imaging (MRI) is the preferred modality. It can diagnose infectious as well as non-infectious etiologies like drug toxicities, metabolic disorders as well as the progression of the disease and response to the therapy.^72,73^ Empiric broad-spectrum antimicrobial therapy including viral and fungal infections are preferred. It is preferred to use empirical bactericidal or fungicidal agents having CNS penetration until a diagnosis is achieved.^71^ There has been always a risk of donor-derived infections in SOT recipients thus donors should be screened with standard screening tests.^74,75^

**Table 4:** Common pathogens causing CNS Infections in SOT recipients

*Intracerebral abscess/space occupying lesion* Bacterial: Embolic or contiguous disease from the local site Nocardia; *Listeria monocytogenes* Fungal: *Aspergillus; Zygomycetes; Cryptococcus* EBV associated posttransplant lymphoproliferative disorder (PTLD) Tuberculosis Toxoplasmosis *Meningoencephalitis* Bacterial: *S. pneumoniae; Neisseria meningitides; Listeria* Viral: CMV; EBV; HSV; VZV; HHV6; Enterovirus; JC virus Fungal: *Cryptococcus, Coccidioides, Histoplasma capsulatum* Tuberculosis Treponema pallidum; Borrelia burgdorferi

## Nocardia: Post transplantation

- We recommend the following regimens for treatment of post-transplant nocardia infections
  1. *Pulmonary*: TMP-SMX (I, A)
  2. *Disseminated or CNS, Critically ill*: Imipenem plus TMP-SMX or Amikacin (I, A)
  3. *Alternative*: Meropenem, Linezolid (I, A)

## Evidence Summary

SOT recipients are at risk of developing nocardia infection which is an opportunistic event.^76^ The risk of developing nocardiosis after SOT varies with the type of organ transplanted, e.g., the highest incidence in recipients of a lung transplant. A review of 5126 organ transplant recipients has demonstrated that highest nocardial infection rate among lung transplant recipients (3.5%).^77^ TMP-SMX is the treatment of choice for nocardial infections as it has demonstrated clinical efficacy and achieves high tissue concentrations in lung, brain, skin, and bone. Combination therapy is recommended in critically ill patients with pulmonary nocardia, cerebral nocardia, and disseminated nocardia.^78^ Linezolid has shown good activity against all species of nocardia.^79^

## References

[1] Fishman JA. (2007;). Infection in Renal Transplant Recipients.. Semin Nephrol..

[2] Al-Hasan MN,, Razonable RR,, Eckel-Passow JE,, Baddour LM. (2009;). Incidence rate and outcome of gram-negative bloodstream infection in solid organ transplant recipients.. Am J Transplant..

[3] Ram R,, Dakshin Murty K,, Prasad N. (2005;). Time table of infections after renal transplantation – SouthIndian experience.. Indian J Nephrol Indian J Nephrol..

[4] Sriperumbuduri S,, Kalidindi K,, Guditi S,, Taduri G. (2017;). Declining trend of infections in renal transplant recipients in a tertiary care hospital from India.. Indian J Transplant.

[5] Kumar A,, Agarwal C,, Hooda AK,, Ojha A,, Dhillon M,, Hari Kumar K.: (2016;). J Family Med Prim Care.

[6] Wang ZX,, Song SH,, Teng F,, Wang GH,, Guo WY,, Shi XM,, Ma J, (2010;). Clin Transplant..

[7] Giannella M,, Muñoz P,, Alarcón JM,, Mularoni A,, Grossi P,, Bouza E;, PISOT study group. (2014;). Pneumonia in solid organ transplant recipients: a prospective multicenter study.. Transpl Infect Dis..

[8] Dizdar OS,, Ersoy A,, Akalin H. (2014;). Pneumonia after kidney transplant: Incidence, risk factors, and mortality.. Exp Clin Transplant..

[9] Hoyo I,, Linares L,, Cervera C,, Almela M,, Marcos MA,, Sanclemente G,, Cofán F, (2010;). Epidemiology of pneumonia in kidney transplantation.. Transplant Proc..

[10] Cisneros JM,, Muñoz P,, Torre-Cisneros J,, Gurgui M,, Rodriguez-Hernandez MJ,, Aguado JM, (1998;). Pneumonia after heart transplantation: a multi-institutional study. Spanish Transplantation Infection Study Group.. Clin Infect Dis..

[11] Lenner R,, Padilla ML,, Teirstein AS,, Gass A,, Schilero GJ. (2001;). Chest.. Pulmonary complications in cardiac transplant recipients..

[12] Atasever A,, Bacakoglu F,, Uysal FE,, Nalbantgil S,, Yagdi T,, Guzelant A,, Sayiner A. (2006;). Pulmonary complications in heart transplant recipients. Transplant Proc..

[13] Lebeaux D,, Freund R,, Van Delden C,, Guillot H,, Marbus SD,, Matignon M,, Van Wijngaerden E, (2017;). Outcome and Treatment of Nocardiosis after Solid Organ Transplantation: New Insights from a European Study.. Clin Infect Dis..

[14] Peleg AY,, Husain S,, Qureshi ZA,, Silveira FP,, Sarumi M,, Shutt KA,, Kwak EJ, (2007;). Risk factors, clinical characteristics, and outcome of Nocardia infection in organ transplant recipients: A matched case-control study.. Clin Infect Dis.

[15] Cervera C,, van Delden C,, Gavaldà J,, Welte T,, Akova M,, Carratalà J. (2014;). Multidrug-resistant bacteria in solid organ transplant recipients.. Clin Microbiol Infect..

[16] Len O,, Rodríguez-Pardo D,, Gavaldã J,, Aguado JM,, Blanes M,, Borrell N,, Bou G, (2012;). Outcome of Clostridium difficile-associated disease in solid organ transplant recipients: A prospective and multicentre cohort study.. Transpl Int..

[17] Haidar G,, Singh N. (2017;). Viral infections in solid organ transplant recipients.. Current Opinion in Infectious Diseases.

[18] Razonable RR,, Humar A. (2013;). Cytomegalovirus in Solid Organ Transplantation.. American Journal of Transplantation..

[19] Lufft V,, Kliem V,, Behrend M,, Pichlmayr R,, Koch KM,, Brunkhorst R. (1996;). Incidence of Pneumocystis carinii pneumonia after renal transplantation. Impact of immunosuppression.. Transplantation..

[20] Borstnar S,, Lindic J,, Tomazic J,, Kandus A,, Pikelj A,, Prah J,, Skvarc M, (2013;). Pneumocystis jirovecii pneumonia in renal transplant recipients: a national center experience. Transplant Proc..

[21] Hayes MJ,, Torzillo PJ,, Sheil AG,, McCaughan GW. (1994;). Pneumocystis carinii pneumonia after liver transplantation in adults. Clin Transplant..

[22] Choi YI,, Hwang S,, Park GC,, Namgoong JM,, Jung DH,, Song GW,, Ha TY, (2013;). Clinical outcomes of Pneumocystis carinii pneumonia in adult liver transplant recipients. Transplant Proc..

[23] Henao-Martínez, A. F.,, Beckham, J. D. (2015;). Cryptococcosis in solid organ transplant recipients.. Current Opinion in Infectious Diseases.

[24] Rakvit A,, Meyerrose G,, Vidal AM,, Kimbrough RC,, Sarria JC. (2005;). Cellulitis caused by Cryptococcus neoformans in a lung transplant recipient.. J Heart Lung Transplant.

[25] Singh N,, Lortholary O,, Alexander BD,, Gupta KL,, John GT,, Pursell K,, Munoz P, (2005;). An immune reconstitution syndrome-like illness associated with Cryptococcus neoformans infection in organ transplant recipients.. Clin Infect Dis.

[25a] Legris T,, Massad M,, Purgus R,, Vacher-Coponat H,, Ranque S,, Girard N,, Berland Y,, Moal V. (2011;). Immune reconstitution inflammatory syndrome mimicking relapsing Cryptococcal meningitis in a renal transplant recipient.. Transplant Infect Dis.

[26] Hsu RB,, Chang CI,, Fang CT,, Chang SC,, Wang SS,, Chu SH. (2011;). Bloodstream infection in heart transplant recipients: 12-year experience at a university hospital in Taiwan.. Eur J Cardiothorac Surg.

[27] Chou NK,, Chi NH,, Wu IW,, Huang SC,, Chen YS,, Yu HY,, Tsao CI, (2010;). Fungal infection in heart transplant recipients with severe sepsis: single-center experience.. Transplant Proc.

[28] Baer S,, Baddley JW,, Gnann JW,, Pappas PG. (2009;). Cryptococcal disease presenting as necrotizing cellulitis in transplant recipients.. Transpl Infect Dis.

[29] Wu G,, Vilchez RA,, Eidelman B,, Fung J,, Kormos R,, Kusne S. (2002;). Cryptococcal meningitis: an analysis among 5521 consecutive organ transplant recipients.. Transpl Infect Dis.

[30] Singh N,, Paterson DL. (2005;). Aspergillus infections in transplant recipients.. Clin Microbiol Rev.

[31] Fortun J,, Martin-Davila P,, Moreno S,, Barcena R,, de Vicente E,, Honrubia A,, Garcia M, (2003;). Prevention of invasive fungal infections in liver transplant recipients: the role of prophylaxis with lipid formulations of amphotericin B in high-risk patients.. J Antimicrob Chemotherap.

[32] Iversen M,, Burton CM,, Vand S,, Skovfoged L,, Carlsen J,, Milman N,, Andersen CB, (2007;). Eur J Clin Microbiol Infect Dis..

[33] Herbrecht R,, Denning DW,, Patterson TF,, Bennett JE,, Greene RE,, Oestmann JW,, Kern WV, (2002;). Voriconazole versus amphotericin B for primary therapy of invasive aspergillosis.. N Engl J Med.

[34] Fortun J,, Martín-Dávila P,, Sánchez MA,, Pintado V,, Alvarez ME,, Sánchez-Sousa A,, Moreno S. (2003;). Voriconazole in the treatment of invasive mold infections in transplant recipients.. Eur J Clin Microbiol Infect Dis..

[35] Denning DW,, Ribaud P,, Milpied N,, Caillot D,, Herbrecht R,, Thiel E,, Haas A, (2002;). Efficacy and safety of voriconazole in the treatment of acute invasive aspergillosis.. Clin Infect Dis.

[36] Veroux M,, Corona D,, Gagliano M,, Sorbello M,, Macarone M,, Cutuli M,, Giuffrida G, (2007;). Voriconazole in the treatment of invasive aspergillosis in kidney transplant recipients.. Transplant Proc.

[37] Schwartz S,, Ruhnke M,, Ribaud P,, Corey L,, Driscoll T,, Cornely OA,, Schuler U, (2005;). Improved outcome in central nervous system aspergillosis, using voriconazole treatment.. Blood.

[38] Wéclawiak H,, Garrouste C,, Kamar N,, Linas MD,, Tall P,, Dambrin C,, Durand D, (2007;). Aspergillus fumigatus related spondylodiscitis in a heart transplant patient successfully treated with voriconazole.. Transplant Proc.

[39] Wieland T,, Liebold A,, Jjagiello M,, Retzl G,, Birnbaum DE. (2005;). Superiority of voriconazole over amphotericin b in the treatment of invasive aspergillosis after heart transplantation.. J heart lung transplant.

[40] Yi WM,, Schoeppler KE,, Jaeger J,, Mueller SW,, MacLaren R,, Fish DN,, Kiser TH. (2017;). Voriconazole and posaconazole therapeutic drug monitoring: A retrospective study.. Annals of Clinical Microbiology and Antimicrobials.

[41] Troke PF,, Hockey HP,, Hope WW. (2011;). Observational study of the clinical efficacy of voriconazole and its relationship to plasma concentrations in patients.. Antimicrob Agents Chemother..

[42] Conti DJ,, Freed BM,, Singh TP,, Gallichio M,, Gruber SA,, Lempert N. (1995;). Preemptive Ganciclovir Therapy in Cytomegalovirus-Seropositive Renal Transplants Recipients.. Arch Surg..

[43] Asberg A1,, Humar A,, Rollag H,, Jardine AG,, Mouas H,, Pescovitz MD,, Sgarabotto D, (2007;). Oral valganciclovir is noninferior to intravenous ganciclovir for the treatment of cytomegalovirus disease in solid organ transplant recipients.. Am J Transplant.

[44] Hibberd PL,, Tolkoff-Rubin NE,, Conti D,, Stuart F,, Thistlethwaite JR,, Neylan JF,, Snydman DR, (1995;). Preemptive ganciclovir therapy to prevent cytomegalovirus disease in cytomegalovirus antibody-positive renal transplant recipients. A randomized controlled trial.. Ann Intern Med.

[45] Marchetti S,, Santangelo R,, Manzara S,, D'Onghia S,, Fadda G,, Cattani P. (2011;). Comparison of real-time PCR and pp 65 antigen assays for monitoring the development of cytomegalovirus disease recipients of solid organ and bone marrow transplants.. New Microbiol.

[46] Humar A,, Kumar D,, Boivin G,, Caliendo AM. (2002;). Cytomegalovirus (CMV) virus load kinetics to predict recurrent disease in solid-organ transplant patients with cmv disease.. J infect dis.

[47] Sia IG,, Wilson JA,, Groettum CM,, Espy MJ,, Smith TF,, Paya CV. (2000;). Cytomegalovirus (CMV) DNA Load Predicts Relapsing CMV Infection after Solid Organ Transplantation.. J Infect Dis..

[48] Asberg A,, Humar A,, Jardine AG,, Rollag H,, Pescovitz MD,, Mouas H,, Bignamini A, (2009;). Long-term outcomes of cmv disease treatment with valganciclovir versus iv ganciclovir in solid organ transplant recipients.. Am J Transplant.

[49] Razonable RR,, Åsberg A,, Rollag H,, Duncan J,, Boisvert D,, Yao JD,, Caliendo AM, (2013;). Virologic suppression measured by a cytomegalovirus (CMV) DNA test calibrated to the world health organization international standard is predictive of CMV disease resolution in transplant recipients.. Clin Infect Dis.

[50] Martin SI,, Fishman JA;, AST Infectious Diseases Community of Practice. (2013;). Pneumocystis pneumonia in solid organ transplantation. Am. J.. Transplant..

[51] Klein NC,, Duncanson FP,, Lenox TH,, Forszpaniak C,, Sherer CB,, Quentzel H,, Nunez M (1992;). Trimethoprim-sulfamethoxazole versus pentamidine for Pneumocystis carinii pneumonia in AIDS patients: Results of a large prospective randomized treatment trial.. AIDS.

[52] O'Brien JG,, Dong BJ,, Coleman RL,, Gee L,, Balano KB. (1997;). A 5-year retrospective review of adverse drug reactions and their risk factors in human immunodeficiency virus-infected patients who were receiving intravenous pentamidine therapy for Pneumocystis carinii pneumonia. Clin. Infect.. Dis..

[53] Benfield T,, Atzori C,, Miller RF,, Helweg-Larsen J. (2008;). Second-line salvage treatment of AIDS-associated Pneumocystis jirovecii pneumonia: A case series and systematic review.. J Acquir Immune Defic Syndr..

[54] Kim T,, Hong HL,, Lee YM,, Sung H,, Kim SH,, Choi SH,, Kim YS, (2013;). Is Caspofungin really an effective treatment for Pneumocystis jirovecii pneumonia in immunocompromised patients without human immunodeficiency virus infection? Experiences at a single center and a literature review. Scand. J. Infect.. Dis..

[55] Fishman JA. (1998;). Treatment of infection due to Pneumocystis carinii.. Antimicrob Agents Chemother..

[56] Roux A,, Gonzalez F,, Roux M,, Mehrad M,, Menotti J,, Zahar JR,, Tadros VX, (2014;). Update on pulmonary Pneumocystis jirovecii infection in non-HIV patients.. Med Mal Infect..

[57] Ewald H,, Raatz H,, Boscacci R,, Furrer H,, Bucher HC,, Briel M. (2015;). Adjunctive corticosteroids for Pneumocystis jirovecii pneumonia in patients with HIV-infection.. Cochrane Database Syst Rev..

[58] Catherinot E,, Lanternier F,, Bougnoux ME,, Lecuit M,, Couderc LJ,, Lortholary O. (2010;). Pneumocystis jirovecii Pneumonia.. Infect Dis Clin North Am..

[59] Bollée G,, Sarfati C,, Thiéry G,, Bergeron A,, de Miranda S,, Menotti J,, de Castro N, (2007;). Clinical picture of Pneumocystis jirovecii pneumonia in cancer patients.. Chest.

[60] Pareja JG,, Garland R,, Koziel H. (1998;). Use of adjunctive corticosteroids in severe adult non-HIV Pneumocystis carinii pneumonia.. Chest.

[61] Subramanian AK,, Morris MI,, AST Infectious Diseases Community of Practice. (2013;). Mycobacterium tuberculosis infections in solid organ transplantation.. Am J Transplant.

[62] Torre-Cisneros J,, Doblas A,, Aguado JM,, San Juan R,, Blanes M,, Montejo M,, Cervera C,, Spanish Network for Research in Infectious Diseases. (2009;). Tuberculosis after solid-organ transplant: incidence, risk factors, and clinical characteristics in the RESITRA (Spanish Network of Infection in Transplantation) cohort,. Clin Infect Dis..

[63] Muñoz P,, Rodríguez C,, Bouza E. (2005;). Mycobacterium tuberculosis infection in recipients of solid organ transplants.. Clin Infect Dis.

[64] Subramanian AK. (2014;). Tuberculosis in solid organ transplant candidates and recipients: current and future challenges.. Curr Opin Infect Dis.

[65] Horne DJ,, Narita M,, Spitters CL,, Parimi S,, Dodson S,, Limaye AP. (2013;). Challenging issues in tuberculosis in solid organ transplantation.. Clin Infect Dis.

[66] Cervera C,, van Delden C,, Gavaldà J,, Welte T,, Akova M,, Carratalà J;, ESCMID Study Group for Infections in Compromised Hosts. (2014;). Multidrug-resistant bacteria in solid organ transplant recipients.. Clin Microbiol Infect..

[67] Zar FA,, Bakkanagari SR,, Moorthi KM,, Davis MB. (2007;). A comparison of vancomycin and metronidazole for the treatment of clostridiumdifficile-associated diarrhea, stratified by disease severity.. Clin Infect Dis..

[68] Fekety R,, Silva J,, Kauffman C,, Buggy B,, Deery HG. (1989;). Treatment of antibiotic-associated clostridium difficile colitis with oral vancomycin: comparison of two dosage regimens.. Am J Med..

[69] Hecht DW,, Galang MA,, Sambol SP,, Osmolski JR,, Johnson S,, Gerding DN. (2007;). In vitro activities of 15 antimicrobial agents against 110 toxigenic clostridium difficile clinical isolates collected from 1983 to 2004.. Antimicrob Agents Chemother..

[70] Kelly CR,, Ihunnah C,, Fischer M. (2017;). Fecal Microbiota Transplant for Treatment of Clostridium difficile Infection in Immunocompromised Patients.. Am J Gastroenterol..

[71] Wright AJ,, Fishman JA. (2014;). Central nervous system syndromes in solid organ transplant recipients.. Clin Infect Dis..

[72] Živković S. (2007;). Neuroimaging and neurologic complications after organ transplantation.. J Neuroimaging..

[73] Fishman JA. (2007;). Infection in Renal Transplant Recipients.. Semin Nephrol.

[74] Grossi PA,, Fishman JA. (2009;). AST Infectious Disease Community of Practice. Donor-derived infections in solid organ transplant recipients.. Am J Transplant.

[75] Ison MG,, Nalesnik MA. (2011;). An update on donor-derived disease transmission in organ transplantation.. Am J Transplant.

[76] Lebeaux D,, Freund R,, Van Delden C,, Guillot H,, Marbus SD,, Matignon M, (2017;). Outcome and Treatment of Nocardiosis after Solid Organ Transplantation: New Insights from a European Study.. Clin Infect Dis..

[77] Peleg AY,, Husain S,, Qureshi ZA,, Silveira FP,, Sarumi M,, Shutt KA,, Kwak EJ, (2007;). Risk factors, clinical characteristics, and outcome of Nocardia infection in organ transplant recipients: A matched case-control study.. Clin Infect Dis.

[78] Clark NM,, Reid GE. (2013;). Nocardia infections in solid organ transplantation.. Am J Transplant..

[79] Moylett EH1,, Pacheco SE,, Brown-Elliott BA,, Perry TR,, Buescher ES,, Birmingham MC,, Schentag JJ, (2003;). Clinical experience with linezolid for the treatment of Nocardia infection.. Clin Infect Dis.

# Article commentary: PART 3. THE HUMAN IMMUNODEFICIENCY VIRUS (HIV) POSITIVE PATIENT IN THE INTENSIVE CARE UNIT

These guidelines are applicable to a patient who is known to be infected with the human immunodeficiency virus (HIV) or presents for the first time to ICU with AIDS-defining conditions.

With the advent of highly active anti retroviral therapy (HAART) era and the Test and Treat policy in HIV (where anyone testing positive receives HAART irrespective of CD4 cell counts). The incidence of ICU admissions for an HIV related illness is decreasing. Although HIV infected patients may still seek intensive care for reasons related directly to HIV infection, more and more seek care for other conditions that are unrelated to HIV such as trauma, infections, and other chronic diseases. Except for some special conditions such as opportunistic infections or HIV related treatment complications, HIV-infected patients are managed similarly to other patients without HIV infection. The treating physician should be aware of drug interactions, infectious and noninfectious conditions as a cause of the clinical presentation.^1^

## The HIV patient in ICU with Acute respiratory failure

## Evidence statements

- Appropriate samples should be collected for staining and cultures–including sputum/induced sputum and bronchoscopic lavage–if indicated. (UPP, A)
- Patients with severe pneumonia who require intensive care and without risk of *Pseudomonas aeruginosa* should be empirically treated with an IV β-lactam plus IV macrolide (II, A). Preferred β-lactams are ceftriaxone, cefotaxime, or amoxicillin-clavulanic acid. In patients who are allergic to penicillin, aztreonam plus azithromycin should be used (III, B).
- Those with CD4 counts < 200/mm^3^ and without signs of focal consolidation may be suspected to have PCP and should receive Trimethoprim-sulphamethoxazole (Co-trimoxazole) in therapeutic dose (TMP 15–20 mg/kg/day plus SMX 75 to 100 mg/kg/day given q6h or q8h). (I, A)
- Patients with documented or suspected Pneumocystis Jerovecii pneumonia (PCP) pneumonia and moderate-to-severe disease, defined by room air PO_2_ < 70 mm Hg or Alveolar-arterial O_2_ gradient ≥ 35 mmHg, should receive adjunctive corticosteroids as early as possible and certainly within 72 hours after starting specific PCP therapy (I, A)
- If patients with HIV/AIDS develop acute respiratory failure and they have any of the risk factors for Pseudomonas infection we recommend dual antipseudomonal coverage such as anti-pseudomonal β-lactam plus aminoglycoside (examples of anti-pseudomonal β-lactams include ceftazidime, cefoperazone, cefoperazone-sulbactam, piperacillin-tazobactam, imipenem-cilastatin, or meropenem (III, A). In patients who are allergic to penicillin, aztreonam can be used in place of the β-lactam. Combination therapy may be considered with the addition of aminoglycosides or antipseudomonal fluoroquinolones (e.g., levofloxacin, ciprofloxacin) (III, B).
- We recommend continuing Azithromycin along with anti-pseudomonal therapy for coverage of atypical pathogens (II, B).
- We recommend against using fluoroquinolones empirically to avoid development of drug-resistant TB. Patients should also undergo sputum testing for acid-fast *bacilli* simultaneously if fluoroquinolones are being used. Fluoroquinolones may be continued only if tuberculosis is not a diagnostic consideration at admission (I, A).
- In patients who have risk factors for methicillin-resistant *Staphylococcus aureus* (MRSA) infection–empiric treatment should include vancomycin or linezolid (III, B).
- We suggest the addition of clindamycin (to vancomycin, but not to linezolid) in cases of severe necrotizing pneumonia to minimize bacterial toxin production (III, B).
- When the etiology of pneumonia has been identified on the basis of reliable microbiological methods, antimicrobial therapy should be de-escalated (II, A).
- A switch to oral therapy should be considered in patients with community acquired pneumonia (CAP) on IV antibiotic therapy who have improved clinically, can swallow and tolerate oral medications, and have intact gastrointestinal function (II, A).

## Evidence Summary

Respiratory failure is the most important cause of ICU admission among HIV patients. However, the specific microbiology data on etiology among HIV positive patients in the ICU is lacking from the Indian sub-continent. After going through the available literature, the most common pathogens seem to be viral infections, *Pneumocystis Jirovecii, Streptococcal pneumoniae, H. Influenzae, M. tuberculosis, Staphylococcus aureus, Klebsiella pneumoniae, Pseudomonas aeruginosa, and Escherichia coli*^2^ (Table 1). These patients are highly susceptible to infections with *Mycobacterium* tuberculosis (MTB). Hence MTB should be actively searched and ruled out in this population, and this may often require invasive interventions like bronchoscopy to get a bronchoalveolar sample.

**Table 1:** Etiology of acute respiratory failure in patients with HIV

*Author/Country*	*Design*	*Study population*	*Microbiology*
Chiang et al Taiwan^3^	Retrospective study	HIV-infected adults admitted to ICU from 2001 to 2010	*P. jirovecii* pneumonia, cytomegalovirus (CMV) pneumonitis
Barbier et al, France^4^	Retrospective study	HIV-infected patients admitted ICU for respiratory failure from 1996 to 2006.	Bacterial pneumonia, *Pneumocystis jirovecii* pneumonia (PCP) Other opportunistic infections
Alves et al Barcelona^5^	Prospectively study	HIV patients in ICU 1993–1998	*Pneumocystis jirovecii* pneumonia (PCP) bacterial Strep and Mtb
Kuan-Yeh Lee Taiwan^6^	Prospectively evaluated	Hospitalized patients July 2009 to March 2012,	Pneumocystis pneumonia–in those with CD4 count <200 cells/mm^3^ In patients with a CD4 count >200 cells/mm^3^–bacterial infections, followed by tuberculosis
Orsini et al Brooklyn^7^	single-center, prospective, and observational study	HIV-infection and respiratory failure admitted to the ICU from December 1, 2011, to February 28, 2013,	bacterial pneumonia *Pneumocystis jirovecii* pneumonia vancomycin-resistant *Enterococcus* (VRE) bacteremia lung abscess

Appropriate sample collection may be taken for staining and cultures (sputum/bronchoscopic lavage or non-directed BAL in intubated patients Table 2). Sample collection should be done as early as possible, preferably within 1 hour and a broad spectrum antibiotic to cover gram-negative organisms may be added empirically. In case there is a delay of collecting the sample, the antibiotics should be administered as fast as possible. Sputum samples should be sent for gram staining, culture, and also special stains for pneumocystis. Cartridge based nucleic acid amplification test (CBNAAT) of samples is beneficial in the early and rapid diagnosis of MTB and multi-drug resistant tuberculosis (MDRTB). In concordance with the surviving sepsis guidelines, we also recommend measuring lactate at baseline.

**Table 2:** Tests On Sputum/Broncho-Alveolar Lavage Fluid

*Cytology*	*Total and differential cell counts*
Microbiology Staining	Gram stain for Bacterial pathogens KOH/calcofluor white preparation for fungal pathogens Ziehl-Neelson for Mycobacteria Special stains such as Silver methenamine for Pneumocystis jerovecii pneumonia
Cultures	Aerobic culture for bacteria, MGIT for *Mycobacterium* tuberculosis, fungal culture
Quantitative or semiquantitative cultures or molecular assays	*Pneumocystis jerovecii* Nocardia *Mycobacterium* tuberculosis *Toxoplasma gondii* *Strongyloides stercoralis* *Legionella pneumophila* *Cryptococcus neoformans* *Histoplasma capsulatum* *Mycoplasma pneumoniae* Influenza A and B viruses Respiratory syncytial virus

**Table 3:** Risk factors for Pseudomonas and Staphylococcal infections

*Risk factors for P. aeruginosa*	*Risk factors for methicillin-resistant S. aureus*
Advanced immunosuppression/full blown AIDS (CD4 count ≤50 cells/mm3) underlying structural lung disease such as bronchiectasis Profound neutropenia Treatment with long term corticosteroids Severely malnourished patients Those residing in nursing homes/health care facilities or who had recent hospitalizations in the last 3 months. Patients on chronic hemodialysis	Recent influenza infection; IV drug abusers Severe, bilateral, necrotizing pneumonia Recent head injury Patients on chronic hemodialysis

The recommendations regarding antibiotic therapy in HIV are based on studies in both ICU and Non-ICU patients admitted with community-acquired pneumonia (Table 4). In a Dutch trial–CAP-START, among patients admitted to inpatient wards for CAP, β-lactam, and macrolide combination therapy was associated with increased mortality – as compared with fluoroquinolone monotherapy. However, the cohort group had a very low incidence of atypical infections. Studies from Switzerland that compared β-lactam monotherapy to combination therapy with a β-lactam plus a macrolide found a non-significant trend of clinical stability in the combination group. A meta-analysis of β-lactam–macrolide combination therapy suggested that the combination was associated with reduced mortality compared with beta-lactam monotherapy. In view of the paucity of literature and unknown efficacy of monotherapy, the expert committee felt that these patients should receive broad-spectrum combination therapy rather than monotherapy.

After starting on initial broad-spectrum antibiotics, once the culture reports are available, the antibiotic therapy should be de-escalated as quickly as possible. Antibiotic de-escalation reduces the possibility of adverse drug effects, treatment costs and reduces the incidence of drug resistance. Parenteral antibiotics can be changed to oral route once clinical stability is attained. Suggested criteria for clinical stability include oral temperature < 37.8° C, heart rate < 100 beats/minute, respiratory rate < 24 breaths/minute, systolic blood pressure ≥ 90 mm Hg, and room air oxygen saturation > 90% or partial pressure of oxygen in arterial blood (PaO_2_) > 60 mm Hg. De-escalation is safe in patients hospitalized with CAP including severe CAP.^12-14^ Antibiotics need to be given for a minimum of 5 days of treatment and may be stopped in case patients remain afebrile for 48 to 72 hours and are clinically stable. A longer duration of treatment might be required in case of metastatic foci of infection, or infection with drug-resistant bacteria such as MRSA or drug-resistant gram-negative bacteria.^15,16^

## HIV Positive Patients Presenting with Suspected Bloodstream Infections or Sepsis of Unknown Origin

## Evidence Statements

- In the presence of sepsis or septic shock, we recommend following the surviving sepsis guidelines similar to the management of other patients with sepsis (UPP, A).
- In the absence of septic shock or absence of risk factors for Pseudomonas a monotherapy with a third-generation cephalosporin or a cephalosporin, the β-lactamase inhibitor is sufficient (II, A)
- In more severe disease states, such as in the presence of organ dysfunction or septic shock–a combination of broad-spectrum antibiotics may be used for initial empiric therapy (III, A).
- Combination therapy is discouraged in the absence of ongoing shock (I, B)
- Empiric gram-positive coverage is suggested for those who have risk factors for MRSA (UPP, A)
- Anti-fungal agents may be considered only if there is no clinical improvement or there is clinical deterioration even after 72 hours of appropriate empirical antibiotics therapy and CD4 counts < 200/mm^3^ (II, A).
- We recommend against the use of empirical antifungal therapy (II, A)
- Those with CD4 < 100/mm^3^ are at high risk for disseminated tuberculosis and hence, need to be worked up for tuberculosis – including blood cultures for tuberculosis (I, B).
- We recommend against empirical anti-tubercular therapy (ATT). In cases of proven mycobacteremia, ATT may be started as per national guidelines or in consult with the ID specialist (I, A).
- Lateral Flow urine LipoArabinomannan Assay (LF-LAM) may be used to assist in the diagnosis of TB only in HIV positive adult in-patients with signs and symptoms of TB (pulmonary and/or extrapulmonary) who have a CD4 cell count < 100 cells/mm^3^, or HIV positive patients who are seriously ill regardless of CD4 count or with unknown CD4 count (II, B).
- LF-LAM should not be used as a screening test for TB (III, A).

**Table 4:** Results of comparison of various antibiotic regimen in Community Acquired Pneumonia.

*Author/Country*	*Design*	*Study population*	*Conclusion*
Postma DF, Netherlands^8^	randomized, crossover trial	Hospitalized patients (non-ICU)	Among patients with clinically suspected CAP, empirical treatment with beta-lactam monotherapy was non-inferior a beta-lactam–macrolide combination or fluoroquinolone monotherapy with regard to 90-day mortality.
Lee JS^9^	Review	adults hospitalized with community-acquired pneumonia	A cluster randomized trial demonstrated an absolute adjusted difference of 2.5% in 90-day mortality favoring β-lactam monotherapy over β-lactam plus macrolide combination therapy. Another randomized trial found an absolute difference of 7.6% in the attainment of clinical stability on day 7 favoring β-lactam plus macrolide combination therapy over monotherapy with β lactam. Six observational studies found that β-lactam plus macrolide combination therapy was associated with relative reductions of 26% to 68% in short-term mortality 3 observational studies reported that fluoroquinolone monotherapy was associated with relative reductions of 30% to 43% in mortality compared with β-lactam monotherapy.
Nie W^10^	Meta-analysis	Included four prospective cohort studies and 12 retrospective cohort studies	In comparison with beta-lactam monotherapy, beta-lactam macrolide dual therapy reduces the risk of mortality in patients with CAP
Garin N, Switzerland^11^	multicenter, non blinded, noninferiority, RCT	580 immunocompetent adult patients with moderately severe CAP	In patients hospitalized for moderately severe CAP, β-lactam monotherapy had delayed clinical stability with monotherapy and hence cannot be considered non inferior.

## Evidence Summary

Bloodstream infections are common in AIDS patients. As with respiratory failure cases, most of the data is from western countries (Table 5). Except for blood stream infections (BSI) caused by tuberculosis, the data from India is scarce. The common organisms seem to be non-typhoid *Salmonellae, Streptococcus pneumoniae, Escherichia coli, Staphylococcus aureus*, and coagulase-negative *Staphylococci. M. Tuberculosis* is not rare if CD4 count 100/mm^3^. The antibiotics policy for these patients remains the same as mentioned in international guidelines for sepsis.^17,18^

## HIV positive patient presenting with signs of CNS infection in ICU

## Evidence Statements

- We recommend brain imaging (preferably MRI) and/or fundoscopy for ruling out raised intra cranial pressure (ICP). If possible the opening pressure should be measured, and physical characteristics such as cerebrospinal fluid (CSF) appearance should be noted (UPP, B)
- A lumbar puncture should be performed for microbiological and biochemical assay including gram stain, India ink stain, aerobic culture, protein, sugar, Adenosine Deaminase (ADA) levels, lactate levels, and cells.
- We suggest a detailed workup for Tuberculosis including an assay for CBNAAT, Mycobacteria growth indicator tube (MGIT) culture, CSF analysis, ADA. (UPP, B)
- In immunocompromised host with relevant clinico-radiological findings and CNS symptoms, additional samples may be sent for *T. gondii* (IgG antigen and/or polymerase chain reaction (PCR), cryptococcal antigen, Mycobacterium tuberculosis (PCR), and common viruses such as Epstein-Barr Virus DNA (EBV) and JC Virus (JCV) along with work up for bacterial culture (UPP, A).

**Table 5:** Common organisms isolated from the bloodstream in patients with HIV

*Reference (First Author, Year)*	*Study Location and Time Frame*	*Study Design*	*Primary Inclusion Criteria*	*Main Isolates in HIV Patients (% of Bacterial Isolates in HIV)*
Grant, 1997^19^	Ivory Coast, 1995	Prospective Observational Study	Admission to the infectious disease unit	Nontyphoid salmonella 23 (59), *E. coli* 6 (15), S. pneumoniae 4 (10)
Hung, 1998^20^	Taiwan,1994–1996	Prospective Observational Study	Fever (≥38°C) in HIV-infected patients admitted to AIDS unit	Non typhoid salmonella 24 (80), *S. aureus* 3 (8), *Klebsiella pneumoniae* 2 (5), *E. coli* 2 (5), *P. aeruginosa* 2 (5)
Manfredi, 1999^21^	Italy, 1994–1995, 1997–1998	Retrospective study	HIV patients admitted to the Hospital	Gram-positive catalase-positive cocci
Mayanja, 2010^22^	Rural Uganda, 1996–2007	Cohort study	Fever (≥38°C) with no detectable malaria Parasites	*S. pneumoniae* 68 (43), NTS 42 (26), CoNS 10 (6), *E. coli* 9 (6)
Meremo, 2012^23^	Urban Tanzania, 2011	Prospective Observational Study	Hospital admission with fever (axillary temperature >37.5° C)	Nontyphoid salmonella 12 (75)
Mootsikapun 2007^24^	Urban Thailand, 1996–2001	Retrospective study	Positive blood culture (including fungi and mycobacteria)	NTS 82 (61), S. aureus 13 (20), Enterobacter spp 11 (8)
Muyanja, 2011^25^	Uganda, 1995–2008	Cohort study–	HIV infected	*S. pneumoniae* 103 (42), NTS 66 (27), *E. coli* 18 (7)
VarmaJK, 2010^26^	thailand	prospective–2006–2008	Outpatients diagnosed with HIV regardless of the presence or absence of symptoms or prior suspicion of clinical illness	M.TB 31 (54%), fungi [13 (22%)], and bacteria [9 (16%)].
Gopinath^27^	India 2005–2006	Prospective study	Hospitalized patients with HIV-and suspected tuberculosis	M.TB 30%
Ramachandran^28^	India	Prospective control study	Hospital admission with a diagnosis of HIV	4% MTB

- For *Cryptococcal Meningitis*, a paired sample of both serum and CSF should be sent for *S. Cryptococcal* antigen assay and CSF Cryptococcal antigen (UPP, A).
- In patients presenting with features of raised ICP and intracranial mass lesions, toxoplasma also needs to be considered. We recommend CSF analysis for IgG antibodies against Toxoplasma antibodies as it has a good negative predictive value in ruling out infections (UPP, A).
- For a patient coming to ICU with altered CNS function and suspicion of meningitis, we recommend a third-generation cephalosporin- known to penetrate the blood-brain barrier - at higher doses, e.g., Ceftriaxone 2 gm BD intravenously (I, A).
- We suggest the addition of vancomycin empirically to the initial treatment regime (I, B).
- We recommend de-escalating antibiotics after culture reports are available (I, A).
- In patients above 50 years of age, we suggest the use of additional ampicillin at high doses of 2 gm every 6th hourly (I, B).
- In very young infants of age < 1 month, we suggest Ampicillin plus cefotaxime or ampicillin plus an aminoglycoside as the initial management (I, B).
- For *Cryptococcal meningitis,* we recommend induction therapy followed by consolidation therapy for 2 months. In patients with severe disease, and low CD4 counts we recommend maintenance therapy for atleast a year (II, A).
- The preferred induction therapy recommended is Liposomal amphotericin B (3 to 5 mg/kg IV daily) plus flucytosine (100 mg/kg per day orally in four divided doses) for a minimum of two weeks. If flucytosine is not available, fluconazole (800 mg daily orally) with Amphotericin B should be given for a minimum of two weeks (I, B).
- In view of the risk of serious adverse effects, we suggest the use of liposomal amphotericin B instead of amphotericin deoxycholate, if cost is not an issue (II, B).
- We recommend Amphotericin B deoxycholate (0.7 - 1 mg/kg/day IV daily) be used if liposomal amphotericin is not available and the risk of renal dysfunction is low. (I, A)
- Consolidation therapy with fluconazole at a dose of 400 mg orally daily for a minimum of eight weeks is recommended (I, A).
- Maintenance therapy: At the completion of eight weeks, fluconazole 200 mg daily should be continued for long-term suppression for a minimum of one year, if CD4 count >100 cells/mm^3^ (II, B).
- Secondary prophylaxis should be reinitiated if the CD4 count decreases again to < 100 cells/mm^3^ (III, A)
- Corticosteroids and mannitol have been shown to be ineffective in managing ICP, and we recommend against the routine use of these agents in cryptococcal meningitis (III, A). In the case of IRIS, corticosteroids should be administered to manage severe central nervous system immune reconstitution inflammatory syndromes. (I, A)

Immediate ART in the setting of *Cryptococcal meningitis* may increase the risk of serious IRIS, a short delay (4–8 weeks) before initiating ART may be necessary.^29,30^

## Empiric therapy for suspected CNS toxoplasmosis

### Evidence Statements

- We recommend a combination of pyrimethamine plus sulfadiazine plus leucovorin in patients with suspected toxoplasmosis (Sulfadiazine 1000 mg four times daily among patients < 60 kg or 1500 mg four times daily among patients ≥ 60 kg, Pyrimethamine 200 mg loading dose followed by 50 mg daily among patients < 60 kg or 75 mg daily among patients ≥ 60 kg, Leucovorin 10 to 25 mg daily) (I, A).
- Pyrimethamine plus clindamycin plus leucovorin is the preferred alternative regimen for patients with TE who cannot tolerate sulfadiazine or do not respond to first-line therapy (I, A).
- If pyrimethamine is unavailable or there is a delay in obtaining it, TMP-SMX should be utilized in place of pyrimethamine-sulfadiazine or pyrimethamine-clindamycin (I, B).
- Acute therapy for Toxoplasmosis should be continued for at least 6 weeks if there is a clinical and radiologic improvement (II, B).
- Adjunctive corticosteroids such as dexamethasone may be administered to patients to treat a mass effect associated with focal lesions or associated edema (III, B)

## Management of Tuberculous meningitis or CNS Tuberculoma

- The management remains the same as in CNS tuberculosis in an immunocompetent individual with 2 months of an intensive regimen with four drugs and 7 to 9 months of continuation phase (total 9–11 months) (I, A)
- If not already on ART, ART should be initiated after 8 weeks of intensive phase, regardless of CD4 count (I, A).
- Systemic corticosteroids are indicated to prevent/manage IRIS. Initiate 0.5 to 1 mg/kg of prednisone daily, and then taper the dose over two to six weeks. (I, A) A good alternative is to administer dexamethasone, tapered over 2 to 6 weeks. (I, A)
- We suggest initiation of antituberculous therapy on the basis of strong clinical suspicion of CNS tuberculosis. Treatment should not be delayed until proof of infection has been obtained (I, B).

## Evidence Summary

Management of treatment-naïve HIV-infected patients with TB is especially challenging in areas with high rates of coinfection. Initiation of antiretroviral therapy may be complicated by the immune reconstitution inflammatory syndrome (IRIS), which can manifest as reactivation of latent TB, the progression of active TB disease, or clinical deterioration in patients previously improving on antituberculous therapy.

The most important factor in determining the differential diagnosis is the degree of immunosuppression in the patient.

In patients with CD4 cell counts > 500/mm^3^, benign and malignant brain tumors and metastases predominate, as in immunocompetent hosts.

In moderately immunosuppressed patients with CD4 cell counts from 200 to 500/mm^3^, HIV-associated cognitive and motor disorders are common but usually, do not present with focal lesions.

In severely immunosuppressed patients with CD4 cell counts < 200/mm^3^, CNS mass lesions are most common. The most likely diagnostic considerations include opportunistic infections (Toxoplasma encephalitis, primary CNS lymphoma, progressive multifocal leukoencephalopathy, HIV encephalopathy, and CMV encephalitis) and AIDS-associated tumors, such as primary central nervous system lymphoma.

In addition, multiple etiologies can coexist in an immunosuppressed individual.^31-33^

Patients who are not under medical supervision, or those who are not aware of their HIV status, may present with their first opportunistic infection in the central nervous system. The clinical manifestations and the diagnostic possibilities are similar to those seen in before the introduction of potent antiretroviral therapy (ART) era. However, the effect of prophylaxis for Pneumocystis and the use of ART may alter the clinical spectrum of disease and the diagnostic considerations in patients who are taking medications.^34-35^ In a patient presenting with CNS symptoms/altered mentation, noninfectious and infectious causes need to be ruled out. A practical approach is shown in the flowchart.



Drug interactions need to be considered when starting treatment of an HIV patient already on HAART with drugs such rifampicin and clarithromycin. Drug resistance is an important factor that has to be considered in these patients hence whenever possible, the expert committee feels that treatment of suspected TB should be initiated only after a microbiological diagnosis and in liaison with an infectious disease expert.

## References

[1] Ganesan A,, Masur H. (2013;). Critical care of persons infected with the human immunodeficiency virus.. Clin Chest Med..

[2] Di Pasquale MF,, Sotgiu G,, Gramegna A,, Radovanovic D,, Terraneo F,, Reyes LF,, Rupp J, Prevalence and Etiology of Community-acquired Pneumonia in Immunocompromised Patients.. https://academic.oup.com/cid/advance-article/doi/10.1093/cid/ciy723/5078600-last.

[3] Chiang HH,, Hung CC,, Lee CM,, Chen HY,, Chen MY,, Sheng WH,, Hsieh SM. (2011;). Admissions to intensive care unit of HIV-infected patients in the era of highly active antiretroviral therapy: etiology and prognostic factors.. Crit Care..

[4] Barbier F,, Coquet I,, Legriel S,, Pavie J,, Darmon M,, Mayaux J,, Molina JM, (2009;). Etiologies and outcome of acute respiratory failure in HIV-infected patients.. Intensive Care Med..

[5] Alves C,, Nicolás JM,, Miró JM,, Torres A,, Agustì C,, Gonzalez J,, Raño A, (2001;). Reappraisal of the aetiology and prognostic factors of severe acute respiratory failure in HIV patients.. Eur Respir J..

[6] Lee KY,, Ho CC,, Ji DD,, Lee CM,, Tsai MS,, Cheng AC,, Chen PY, (2013;). Etiology of pulmonary complications of human immunodeficiency virus-1-infected patients in Taiwan in the era of combination antiretroviral therapy: a prospective observational study.. J Microbiol Immunol Infect..

[7] Orsini J,, Ahmad N,, Butala A,, Flores R,, Tran T,, Llosa AFE. Etiology and Outcome of Patients with HIV Infection and Respiratory Failure Admitted to the Intensive Care Unit.. Interdiscip Perspect Infect Dis..

[8] Postma DF,, van Werkhoven CH,, van Elden LJ,, Thijsen SF,, Hoepelman AI,, Kluytmans JA,, Boersma WG, (2015;). BMC-SSG. Antibiotic Treatment Strategies for Community-Acquired Pneumonia in Adults.. N Engl J Med..

[9] Lee JS,, Giesler DL,, Gellad WF,, Fine MJ. (2016;). Antibiotic Therapy for Adults Hospitalized With Community-Acquired Pneumonia.. JAMA..

[10] Nie W,, Li B,, Xiu Q. (2014;). β-Lactam/macrolide dual therapy versus β-lactam monotherapy for the treatment of community-acquired pneumonia in adults: a systematic review and meta-analysis.. J Antimicrob Chemother..

[11] Garin N,, Genné D,, Carballo S,, Chuard C,, Eich G,, Hugli O,, Lamy O, (2014;). β-Lactam monotherapy vs β-lactam-macrolide combination treatment in moderately severe community-acquired pneumonia: a randomized noninferiority trial.. JAMA Intern Med..

[12] Viasus D,, Simonetti AF,, Garcia-Vidal C,, Niubó J,, Dorca J,, Carratalà J. (2017;). Impact of antibiotic de-escalation on clinical outcomes in community-acquired pneumococcal pneumonia.. J Antimicrob Chemoth..

[13] Carugati M,, Franzetti F,, Wiemken T,, Kelly R,, Peyrani P,, Blasi F,, Ramirez J (2015;). De-escalation therapy among bacteraemic patients with community-acquired pneumonia.. Clin Microbiol Infect..

[14] Yamana H,, Matsui H,, Tagami T,, Hirashima J,, Fushimi K,, Yasunaga H. (2016;). De-escalation versus continuation of empirical antimicrobial therapy in community-acquired pneumonia.. J Infect..

[15] Uranga A,, España PP,, Bilbao A,, Quintana JM,, Arriaga I,, Intxausti M, (2016;). Duration of Antibiotic Treatment in Community-Acquired Pneumonia.. JAMA Intern Med..

[16] Mandell LA,, Wunderink RG,, Anzueto A,, Bartlett JG,, Campbell GD,, Dean NC,, Dowell SF, (2007;). Infectious Diseases Society of America/American Thoracic Society Consensus Guidelines on the Management of Community-Acquired Pneumonia in Adults.. Clin Infect Dis..

[17] Rhodes A,, Evans LE,, Alhazzani W,, Levy MM,, Antonelli M,, Ferrer R,, Kumar A, (2017;). Surviving Sepsis Campaign: International Guidelines for Management of Sepsis and Septic Shock: 2016.. Intensive Care Med..

[18] Levy MM,, Evans LE,, Rhodes A. (2018;). The Surviving Sepsis Campaign Bundle: 2018 Update.. Crit Care Med..

[19] Grant AD,, Djomand G,, Smets P,, Kadio A,, Coulibaly M,, Kakou A,, Maurice C, (1997;). Profound immunosuppression across the spectrum of opportunistic disease among hospitalized HIV-infected adults in Abidjan, Côte d'Ivoire.. AIDS..

[20] Hung CC,, Hsueh PR,, Hsieh SM,, Liu CJ,, Chen MY,, Luh KT. (1998;). Bacteremia and fungemia in patients with advanced human immunodeficiency virus (HIV) infection in Taiwan.. J Formos Med Assoc..

[21] Manfredi R,, Nanetti A,, Ferri M,, Chiodo F. (1999;). HIV-associated non-mycobacterial sepsis-bacteraemia, before and during the highly active antiretroviral therapy era.. AIDS..

[22] Mayanja BN,, Todd J,, Hughes P,, Van der Paal L,, Mugisha JO,, Atuhumuza E,, Tabuga P, (2010;). Septicaemia in a population-based HIV clinical cohort in rural Uganda, 1996-2007: incidence, aetiology, antimicrobial drug resistance and impact of antiretroviral therapy.. Trop Med Int Heal..

[23] Meremo A,, Mshana SE,, Kidenya BR,, Kabangila R,, Peck R,, Kataraihya JB. (2012;). High prevalence of Non-typhoid salmonella bacteraemia among febrile HIV adult patients admitted at a tertiary Hospital, North-Western Tanzania.. Int Arch Med..

[24] Mootsikapun P. (2007;). Bacteremia in adult patients with acquired immunodeficiency syndrome in the northeast of Thailand.. Int J Infect Dis..

[25] Muyanja SZ,, Larke N,, Rutebarika D,, Kaddu I,, Nakubulwa S,, Levin J,, Grosskurth H, (2011;). Decreasing trends of bacteraemia among HIV-infected Ugandan adults: incidence, aetiology, clinical outcomes and effect of antiretroviral therapy in a semi-urban setting (2000-2008).. Trop Med Int Heal..

[26] Varma JK,, McCarthy KD,, Tasaneeyapan T,, Monkongdee P,, Kimerling ME,, Buntheoun E,, Sculier D, (2010;). Bloodstream infections among HIV-infected outpatients, Southeast Asia.. Emerg Infect Dis..

[27] Gopinath K,, Kumar S,, Singh S. (2008;). Prevalence of mycobacteremia in Indian HIV-infected patients detected by the MB/BacT automated culture system Medicinal Orchids of Arunachal Pradesh; Micro propagation, Phytochemical Investigation and Biological Activity Evaluation View project.. Eur J Clin Microbiol..

[28] Ramachandran R,, Swaminathan S,, Somasundaram S,, Asgar VN PP,, Paramasivan CN. (2002;). Mycobacteremia in tuberculosis patients with HIV infection.. Ind J Tubrculosis..

[29] Boulware DR,, Meya DB,, Muzoora C,, Rolfes MA,, Huppler Hullsiek K,, Musubire A,, Taseera K, (2014;). Timing of antiretroviral therapy after diagnosis of cryptococcal meningitis.. N Engl J Med..

[30] Panel on Antiretroviral Guidelines for Adults and Adolescents. Guidelines for the Use of Antiretroviral Agents in Adults and Adolescents Living with HIV..

[31] Stenzel W,, Pels H,, Staib P,, Impekoven P,, Bektas N,, Deckert M. (2004;). Concomitant manifestation of primary CNS lymphoma and Toxoplasma encephalitis in a patient with AIDS.. J Neurol.

[32] Gildenberg PL,, Gathe JC, Kim JH. (2000;). Stereotactic biopsy of cerebral lesions in AIDS.. Clin Infect Dis..

[33] Gheuens S,, Cheeseman SH,, Koralnik IJ. (2011;). Hidden in plain view: emergence of progressive multifocal leukoencephalopathy after treatment of CNS toxoplasmosis.. Acta Neurol Belg..

[34] Collazos J. (2003;). Opportunistic Infections of the CNS in Patients with AIDS.. CNS Drugs..

[35] Tan IL,, Smith BR,, von Geldern G,, Mateen FJ,, McArthur JC. (2012;). HIV-associated opportunistic infections of the CNS.. Lancet Neurol..

# Article commentary: PART 4. INDIAN ANTIMICROBIAL PRESCRIPTION GUIDELINES FOR PATIENTS WITH CONGENITAL AND ACQUIRED HYPOSPLENISM/ASPLENIA

Patients with congenital and acquired hyposplenism/asplenia are prone to specific infections and are at increased risk of severe sepsis. Although the incidence of septicemia remains low, the risk for overwhelming post-splenectomy infection (OPSI) remains higher than in the general population.^1^ Majority of the overwhelming post-splenectomy infection (OPSI) is caused by encapsulated bacteria such as Streptococcus pneumonia, Haemophilus influenza, and Neisseria meningitides although other infections can also occur.^2,3^ The disease starts as a minor flu-like illness and rapidly evolves into a fulminant course of hypoglycemia, metabolic acidosis, dyselectrolytemia, disseminated intravascular coagulation (DIC), shock, coma and death within 24 to 48 hours.^4^ OPSI usually occurs within the first two years after splenectomy but may also occur later and has a mortality rate of 50 to 70% despite aggressive therapy.^2^ In view of the severe progression and high mortality of OPSI, stress has been given for early aggressive treatment as well as immunization of patients with splenectomy and thereby preventing OPSI.

## Evidence Statements

- If an asplenic/hyposplenic patient is suspected to have sepsis we recommend administration of IV ceftriaxone before transferring the patient to a higher center (II, A).
- We recommend that all patients with Overwhelming Post-Splenectomy Infection (OPSI) be treated in the ICU. (UPP, A)
- We recommend empiric antibiotic therapy for asplenic patients with a combination of ceftriaxone and vancomycin. (I, A)
- In case of allergy to β-lactams, we recommend vancomycin with aztreonam or fluoroquinolones in adults. Do not delay the administration of antibiotics, be prepared to treat the reaction. (UPP, A)
- We recommend adding clarithromycin or erythromycin in case of respiratory symptoms (III, A).
- We recommend empiric therapy with IV Cefotaxime + vancomycin+ ampicillin if the patient age < 2 months: (III, A)
- All febrile asplenic patients should be screened for malaria with peripheral smears. Start artesunate-based antimalarial therapy, if the history is suggestive of Malaria (UPP, A).
- If gram staining of peripheral blood smear shows gram-negative *bacilli*, we recommend the addition of antipseudomonal coverage to the therapy (III, A).
- We recommend that urine be checked for a urinary antigen for streptococcus pneumonia (II, A).
- We suggest RT-PCR test for simultaneous identification of 3 main encapsulated bacteria (*Strep pneumonia, H. influenzae* type B and *N. meningitidis*) (III, B).
- We recommend that all asplenic patients should receive immunization against encapsulated bacteria (*S. pneumoniae, H. influenzae,* and *N. meningitidis*) (I, A).
- Immunization against seasonal flu is recommended for patients over 6 months of age (I, A).
- We recommend that vaccination programs should be started no sooner than 14 days after splenectomy (I, A).
- If the patient is discharged before 15 days after splenectomy or angioembolization, where the risk to miss vaccination is deemed high, we suggest that patient be vaccinated before discharge (I, B).
- Antibiotic prophylaxis is indicated in patients for 1-2 years after splenectomy and lifelong for the patient had an episode of overwhelming infection or immunocompromised (II, B).
- We recommend self-administration of one dose of, in stock “pill in the pocket”, prescribed antibiotics in the event of any sudden onset of unexplained fever, malaise, chills or other constitutional symptoms, when medical consultation not readily accessible within 2 hours (II, A).
- We suggest that any patient with sepsis having a risk factor for hyposplenia, the peripheral smear should be checked for Howell-Jolly bodies (II, B).
- We recommend formulation of Spleen registry (UPP, A).

## Evidence Summary

Splenectomy is often indicated in patients with an underlying malignant or nonmalignant hematologic diseases or cases of splenic rupture following infection or trauma. Other causes of hyposplenia include auto infarction in subjects with sickle cell anemia and chronic graft-versus-host disease after stem-cell transplantation, severe celiac disease, and untreated human immunodeficiency virus infection.^5^

Overwhelming post-splenectomy infection (OPSI) is defined as an infection, occurring more commonly after splenectomy (or in hyposplenic host) which evolves over a short time and produces severe symptoms, often with hypotension and a high mortality rate.^6^

Patients with hyposplenism due to splenectomy or hyposplenia are at an increased risk for invasive infections with encapsulated bacteria as *Streptococcus pneumoniae*, Haemophilus influenza type b, *Neisseria meningitidis*.^2,7^ These infections progress rapidly from a mild flu-like illness to fulminant sepsis and are associated with a high mortality rate of up to 50% despite maximal treatment. The lifetime risk of OPSI is assumed to be 5%, and the highest frequency of these OPSIs is during the first 2 years after splenectomy.^6-9^ Patients with sickle cell anemia, thalassemia major or malignancies such as Hodgkin's lymphomas and non-Hodgkin's lymphomas have a higher risk for OPSI. Asplenic patients have a higher incidence of parasitemia, a delayed clearance of parasites after treatment or a severe or even fatal infection due to malaria. These patients are also at high risk for Babesiosis, and this might be confused with *Plasmodium falciparum.*^2^ These patients are at an increased risk of OPSI with Capnocytophaga canimorsus, if bitten by dogs and other animals and should receive adequate antibiotic coverage following such bites.^10^ Otherwise rare, Ehrlichiosis is also more severe in patients with asplenia/hyposplenia.^2^

OPSI should be considered as a medical emergency and mandates early recognition and aggressive management. These patients should be managed aggressively including immediate cultures and administration of a combination of antibiotics to cover all possible etiological agents. In areas where penicillin-resistant pneumococci are prevalent, other agents such as vancomycin, teicoplanin or rifampicin should be added to ceftriaxone as the initial empiric therapy. Gram stain of the peripheral blood or buffy coat will give an idea regarding the presence or absence of intraleukocytic bacteria. Antipseudomonal coverage should be added in case of high risk for Pseudomonas infection or peripheral blood growing GNB. The presence of intracellular bacteria within leukocytes should alert the clinician towards ehrlichiosis while the presence of parasites in RBC should alert for malaria or babesiosis. Once the blood cultures are positive antibiotics can be modified accordingly.

## References

[1] Castagnola E,, Fioredda F. (2003;). Prevention of life-threatening infections due to encapsulated bacteria in children with hyposplenia or asplenia: a brief review of current recommendations for practical purposes.. Eur J Haematol..

[2] Sumaraju V,, Smith LG,, Smith SM. (2001;). Infectious complications in asplenic hosts.. Infect Dis Clin North Am..

[3] Davidson RN,, Wall RA. (2001;). Prevention and management of infections in patients without a Spleen.. Clin Microbiol Infect..

[4] Evans DI. (1985;). Postsplenectomy sepsis 10 years or more after operation.. J Clin Pathol..

[5] Leone G,, Pizzigallo E. (2015;). Bacterial Infections Following Splenectomy for Malignant and Nonmalignant Hematologic Diseases.. Mediterr J Hematol Infect Dis..

[6] Morgan TL,, Tomich EB. (2012;). Overwhelming post-splenectomy infection (OPSI): a case report and review of the literature.. J Emerg Med..

[7] Waghorn DJ. (2001;). Overwhelming infection in asplenic patients: current best practice preventive measures are not being followed.. J Clin Pathol..

[8] Brigden MLB. (1992;). Overwhelming postsplenectomy infection still a problem.. West J Med..

[9] Thomsen RW,, Schoonen WM,, Farkas DK,, Riis A,, Jacobsen J,, Fryzek JP,, Sørensen HT. (2009;). Risk for hospital contact with infection in patients with splenectomy: a population based cohort study.. Ann Intern Med..

[10] Sawmiller CJ,, Dudrick SJ,, Hamzi M. (1998;). Postsplenectomy Capnocytophaga canimorsus sepsis presenting as an acute abdomen.. Arch Surg..

# Article commentary: PART 5. PATIENTS WITH PRIMARY IMMUNE DEFICIENCY IN THE INTENSIVE CARE UNIT

Primary immunodeficiencies are a group of disorders that affect the development, function or both of the immune system. There are more than 300 disorders defined till date. The prevalence is approximately 1 in 10,000 live births.^1-3^ Any patient admitted to ICU could be a potential PID patient.

## Diagnosis of PID

### Evidence Statements

- PID should be suspected when the following history/symptoms or signs are present (UPP, A)
  1. Family history of sibling death
  2. Four or more ear infections within 1 year
  3. Two or more serious sinus infections or pneumonias within 1 year
  4. Two or more months on antibiotics with little effect
  5. Two or more deep-seated infections including septicemia
  6. Persistent thrush in mouth or fungal infection on the skin
  7. Infections in multiple anatomic locations
  8. Increasing frequency and severity of infections with age
  9. Recurrent serious infections with common pathogens
  10. Serious infections with unusual pathogens
- We recommend that when PID is suspected, HIV infection should also be considered, and testing should be performed for HIV (UPP, A)
- We recommend that patient should be investigated for PID when:
  1. In neonates, absolute lymphocyte count (ALC) of < 2000/mm^3^ in cord blood or an infant an ALC of < 4000/mm^3^
  2. Severe hypogammaglobulinemia with IgG < 1 50mg/dL.
  3. Absolute lymphocyte count < 4000/mm^3^ (In non-chemotherapy setting)
  4. Unusual organism picked up on microbiology
  5. Unexplained neutropenia
- We recommend that Initial laboratory screening should include a complete blood count with differential counts (including absolute lymphocyte count, absolute neutrophil count, absolute monocyte count) and measurement of serum immunoglobulin and complement levels (UPP, A). We recommend severe combined immune deficiency (SCID) be considered as a pediatric emergency and attention be paid to absolute lymphocyte count, at all time in ICU. If the absolute lymphocyte count is less than normal for the age, we recommend to make immunology reference, use irradiated blood products, and avoid live vaccines till diagnosis is confirmed or ruled out (UPP, A).
- We recommend that patient be investigated for combined variable immuno-deficiency (CVID) when the patient has any of the following: (UPP, A)
  1. Recurrent bacterial infections.
  2. Serum IgG, IgM, IgA levels (at least two of the three) with a marked decrease (at least 2 SD below the mean for age)
  3. The onset of immunodeficiency at more than 2 years of age.
  4. The absence of isohemagglutinins and or poor response to vaccines.
- We recommend that immunology consult be obtained for these patients and the patient be investigated to diagnose specific form of immunodeficiency (UPP, A)
  1. Lymphocyte subpopulations by flow cytometry (CD3, CD4, CD8, CD19, CD20, CD16 and CD56).
  2. Naive T cells, Memory B cells, Memory T cells
  3. T-cell response to mitogens.
  4. Nitroblue tetrazolium-NBT test
  5. Complement levels
  6. Bone marrow and genetic tests

### Evidence Summary

Diagnosis is often delayed since signs and symptoms such as bronchitis, pneumonia, sinusitis, and diarrhea are considered infection related without suspecting immunological process.

The absence of adenoid tissue in the nasopharynx or absence of the thymus should prompt suspicion of primary immunodeficiency (antibody or cellular/combined).

The presence of lymphocytopenia on complete blood count suggests a T-cell disorder, whereas a finding of neutropenia suggests a phagocytic disorder. Abnormal serum immunoglobulin levels suggest a B-cell disorder. Abnormalities on assay of the classic or alternative complement pathways suggest a complement disorder.^4^ Abnormal values of lymphocyte count should also raise suspicion of PID (Table 1).

### Evidence Statement

- We recommend appropriate cultures and PCRs; for organisms likely to cause infections pertinent to the conditions they are suffering from (UPP, A).
- An attempt should be made to identify the microorganisms directly or on PCRs as serological tests in infectious diseases could give false-negative results if there is an antibody defect (UPP, A).
- We recommend the use of Multiplex PCR to help diagnose infections (UPP, A)

**Table 1:** Absolute Lymphocyte count (ALC) nomogram Any value below the reference range should raise suspicion of PID

*Age*	*Lymphocytes (per mm3)*	*Range (per mm3)*
Neonatal	4.8	0.7–7.3
1–2 month	6.7	3.5–13.1
2–5 months	5.9	3.7–9.6
5–9 months	6.0	3.8–9.9
9–15 months	5.5	2.6–10.4
15–24 months	5.6	2.7–11.9
2–5 years	3.3	1.7–6.9
5–10 years	2.8	1.1–5.9
10–16 years	2.2	1.0-5.3
>16 years	1.8	1.0–2.8

### Evidence Summary

Patients with PID commonly present with recurrent infections and invasive infections, atypical pathogens, partial response to antibiotics, failure to thrive, chronic diarrhea, fungal infections, unexplained skin rash, and family history. Infections such as Pneumonia and bronchiolitis, acute gastroenteritis, otitis media, and bacteremia in patients with an antibody, combined, and cellular deficiencies. Whereas viral infections meningitis, osteomyelitis, gastroenteritis is commonly seen in CVID. Children tend to have bacterial or fungal infections with unusual organisms or unusually severe and recurrent infections with common organisms. A family history of primary immunodeficiency disease is the strongest predictor of a person having this type of disease.^5^

The typical presentations of various PIDs by age of presentation and spectrum of infections.

- Combined T-cell and B-cell immunodeficiency (Presents early in life)
  1. *Bacteria*: *Campylobacter Listeria*, Pyogenic bacteria, Mycobacteria
  2. *Viruses*: RSV, EBV, Parainfluenza Virus
  3. *Fungi*: *Candida, Aspergillus*
  4. *Protozoa*: *Pneumocystis jiroveci, Toxoplasma Gondi, Cryptosporidium parvum*
- B cell immunodeficiency (Presents when weaning is started and breastfeeding stops)
  1. *Bacteria*: *S. pneumonia, H. influenza, M. catarrhalis, P. aeruginosa, S. aureus, N. meningitidis, M. pneumonia*
  2. *Viruses*: *Enteroviruses*
  3. *Protozoa*: *Giardia lamblia*
- Congenital defects of phagocyte number and function (Can present at any age based on the severity of the defect)
  1. *Bacteria*: *S.aureus, P. aeruginosa, Nocardia, S. Typhi*
  2. *Fungi*: *Candida, Aspergillus*
  3. *Mycobacteria*: *Nontuberculous* including BCG
- Complement deficiencies (Can present as early as within 6 months of life)
  1. *Bacteria*: *Streptococci, H. influenza, Neisseria,*
  2. *Viruses*: CMV, HSV

The European Society for Immunodeficiency (ESID) clinical guidelines proposed the grouping of immunodeficiency, syndromes and likely infections as follows (Table 2).

**Table 2:** The types of clinical patterns of presentation and infections in PIDs

*Immunodeficiency*	*Infections*	*Example*
Antibody deficiency Phagocyte deficiency Complement deficiency	Bronchiectasis, rhinosinusitis	HIV, Wiskott-Aldrich Syndrome
T-lymphocyte deficiency	Chronic diarrhea, Candida/PCP, Mycobacteria	SCID/HIV
Neutrophil defects	Recurrent pyogenic infections, Invasive *Aspergillus*, Burkholderia	Chronic granulomatous disease (CGD)
Defects of innate immunity(TLR3) T-lymphocyte deficiency	Invasive pneumococcal disease Herpes Simplex Encephalitis	SCID/HIV Wiskott-Aldrich Syndrome
T lymphocyte/macrophage deficiency	*Meningococci*, encapsulated bacteria or *Candida*/Mycobacteria	
Common variable immunodeficiency (CVID)	Autoimmune or chronic inflammatory disease	Haemophagocytic lymphohistiocytosis (HLH)

In ICU setting in patients with PID; following organisms are likely to cause infections.

- B cell deficiency
  1. Pneumococcus
  2. H. influenza
  3. Staph Aureus
  4. Giardia Lamblia
  5. Viruses *Enterovirus/echovirus*
- T cell deficiency
  1. Mycobacteria
  2. *Viruses*: CMV/EBV/HSV/RSV/VZ/Parainfluenza
  3. *Fungi*: *P.carini*, Histoplasma, *cryptosporidium*, Toxoplasma
- Phagocytic disorder
  1. Gram Negative: E.coli/Klebsiella/B.cepacia/Pseudomonas/Serratia
  2. Gram positive: Staph/Nocardia/Listeria/
  3. *Fungus: Aspergillus* and *candida*
- *Defects in the complement system: Streptococcus pneumonia* and *Neisseria*
- *Mendelian susceptibility to Mycobacterium (MSMD): Mycobacteria, salmonella typhi,* and *nontyphii, listeria, viral and other intracellular pathogens* (e.g., Histoplasma, leishmania).^6-11^
- We recommend intravenous immunoglobulin for treatment of all antibody deficiency diseases (UPP, A). The recommended dose is 400 mg/kg/doses every 4 weekly. We recommend 2 gm/kg single dose (severe infections) or 1 gm/kg weekly till infection subsides (UPP, A).
- We recommend to maintain serum IgG trough levels above 500 mg/dL and above 700 mg/dL in bronchiectasis (Level III, A).
- We recommend thoracic computed axial tomography, lung function tests with spirometry and DLCO every 6 months after discharge (UPP, A).
- We recommend hematopoietic stem cell transplantation in cellular and macrophage immunodeficiency (UPP, A).
- We recommend monoclonal antibodies such as rituximab only in autoimmune complications related to CVID (UPP, A).
- We recommend Rituximab be given in PID complicated with EBV viremia (UPP, A).
- We recommend for all critically Ill patients with suspicion of PID the empirical antimicrobial treatment with IV Carbapenems with IV Vancomycin/Teicoplanin for broad-spectrum coverage (UPP, A). Voriconazole is the preferred antifungal in case of proven, possible or probable invasive fungal infection with Aspergillus (I, A).
- In critically ill patients diagnosed with Combined B and T cell deficiency, the antimicrobial drug of choice is IV Carbapenems with Vancomycin/Teicoplanin and Trimethoprim-Sulfamethoxazole (UPP, A).
- In critically ill patients diagnosed with Combined B and T cell deficiency with suspicion of viral infections, we recommend: (UPP, A).
  1. IV Acyclovir if herpes group of infection is suspected
  2. Oral Oseltamivir if Influenza virus is suspected
  3. IV Ganciclovir if CMV is suspected radiologically or by laboratory tests
- In critically ill patients diagnosed with B cell deficiency, based on the organisms expected (Capsulated), we recommend IV ceftriaxone with IV Vancomycin/Teicoplanin (UPP, A).
- We recommend IV immunoglobulin (IVIG) at a dose of 1 gm/kg weekly in cases of severe infections especially ECHO/Enterovirus/Polio virus-induced encephalitis (UPP, A).
- In critically ill patients diagnosed with Phagocyte disorder, we recommend antimicrobial drug of choice to be IV Carbapenems with IV Vancomycin/Teicoplanin and Voriconazole (UPP, A)
- We Recommend the use of Granulocyte-colony stimulating factor (GCSF) in patients of congenital Neutropenia. (UPP, A)
- In critically ill patients diagnosed with complement deficiency, the antimicrobial drug of choice is IV Cephalosporin (UPP, A).

### Evidence Summary

The data regarding the use of Antibiotics in Immunodeficiency states are scarce. The experts recommend using antibiotic as per organism isolated or expected. Generally, the management depends upon the type of PID.

### Therapy includes

- IV Immunoglobulin (IVIG) infusion mainly for B cell deficiency.^12-14^
- Antibiotics as per suspected source of infection and suspected organism
- Rituximab in PID with Epstein Barr virus reactivation

Stem cell transplant is the most curative option for the majority of the PID. Paradoxically Rituximab treatment has known to aggravate primary immunodeficiency or hypogammaglobulinemia in a certain group of patients, and appropriate care has to be taken in these patients. In PID such as X-linked Lymphoproliferative disorder, Rituximab can be given once in 4 weeks to decrease the EBV Viral load.^1,3,4^

## Vaccinations and Antimicrobial Prophylaxis at Discharge

### Evidence Statements

- We wish to emphasize that all forms of live vaccines, viral and bacterial, are contraindicated in patients with SCID (UPP, A).
- We recommend vaccination for diagnosed patients with complement deficiency at the time of discharge (UPP, A). We recommend to avoid BCG vaccination in chronic granulomatous disease/MSMD patient (UPP, A).
- We recommend antifungal, and anti PCP prophylaxis for all patients diagnosed with PID shifted from ICU. (UPP A). PID patients with the chronic granulomatous disease the drug of choice is Itraconazole (I, A) and Trimethoprim-Sulfamethoxazole (II, A).

PCP prophylaxis should be given to all patients with Combined B and T or T cell deficiency with the drug of choice being *Trimethoprim-Sulfamethoxazole* (I, A). We recommend antifungal prophylaxis in all patients with T cell defects (III, A)

### Evidence Summary

Vaccine recommendations should be earmarked only for patients with certain PID.

Live vaccines are avoided in patients with severe B- and T-cell dysfunction due to the risk of dissemination and the futility of immune response. All vaccines are safe and effective in the patients with complement deficiency (susceptibility to encapsulated organisms).^15-17^

## References

[1] Gupta S,, Madkaikar M,, Singh S,, Sehgal S. (2012;). Primary immunodeficiencies in India: a perspective.. Ann N Y Acad Sci..

[2] Jindal AK,, Pilania RK,, Rawat A,, Singh S. (2017;). Primary Immunodeficiency Disorders in India-A Situational Review.. Front Immunol..

[3] Madkaikar M,, Mishra A,, Desai M,, Gupta M,, Mhatre S,, Ghosh K. (2013;). Comprehensive Report of Primary Immunodeficiency; Disorders from a Tertiary Care Center in India.. J Clinical Immunology..

[4] Madkaikar M,, Mishra A,, Ghosh K. (2013;). Diagnostic Approach to Primary Immunodeficiency Disorders;. Indian Pediatrics;.

[5] Halliday E,, Winkelstein J,, Webster AD. (2003;). Enteroviral Infections in Primary Immunodeficiency (PID): A Survey of Morbidity and Mortality;. J Infect..

[6] Antachopoulos C,, Walsh TJ,, Roilides E. (2007;). Fungal infections in primary immunodeficiencies;. Eur J Pediatr..

[7] Ram S,, Lewis LA,, Rice PA. (2010;). Infections of people with complement deficiencies and patients who have undergone splenectomy.. Clin Microbiol Rev..

[8] Odek C,, Kendirli T,, Doğu F,, Yaman A,, Vatansever G,, Cipe F,, Haskoloğlu S, (2014;). Patients with primary immunodeficiencies in pediatric intensive care unit: outcomes and mortality-related risk factors.. J Clin Immunol..

[9] El Habbal MH,, Strobel S. (1993;). Leukocyte adhesion deficiency.. Arch Dis Child.

[10] Bousfiha A,, Jeddane L,, Picard C,, Ailal F,, Bobby Gaspar H,, Al-Herz W,, Chatila T, (2018;). The 2017 IUIS Phenotypic Classification for Primary Immunodeficiencies.. J Clin Immunol..

[11] Abolhassani H,, Sagvand BT,, Shokuhfar T,, Mirminachi B,, Rezaei N,, Aghamohammadi A. (2013;). A review on guidelines for management and treatment of common variable immunodeficiency;. Expert Rev Clin Immunol..

[12] Boyle ML,, Scalchunes C. (2008;). Impact of intervenous immunoglobulin (IVIG) treatment among patients with Primary Immunodeficiency diseases.. Pharmaceuticals Policy and Law.

[13] Shabani M,, Nichols KE,, Rezaei N. (2016;). Primary immunodeficiencies associated with EBV-Induced lymphoproliferative disorders.. Critical Reviews in Oncology/Hematology.

[14] Bonagura VR. (2013;). Using intravenous immunoglobulin (IVIG) to treat patients with primary immune deficiency disease.. J Clin Immunol..

[15] Freeman AF,, Holland SM. (2009;). Antimicrobial prophylaxis for primary immunodeficiencies.. Curr Opin Allergy Clin Immunol..

[16] Rubin LG,, Levin MJ,, Ljungman P,, Davies EG,, Avery R,, Tomblyn M,, Bousvaros A, (2014;). IDSA clinical practice guideline for vaccination of the immunocompromised host.. Clin Infect Dis.

[17] Brady MT,, Bernstein HH,, Byington CL,, Edwards KM,, Fisher MC,, Glode MP,, Jackson MA (2011;). for Committee on Infectious Diseases. Meningococcal conjugate vaccines policy update: booster dose recommendations.. Pediatrics.
